# Inhibition of Glutamate‐to‐Glutathione Flux Promotes Tumor Antigen Presentation in Colorectal Cancer Cells

**DOI:** 10.1002/advs.202310308

**Published:** 2024-10-31

**Authors:** Tao Yu, Kevin Van der Jeught, Haiqi Zhu, Zhuolong Zhou, Samantha Sharma, Sheng Liu, Haniyeh Eyvani, Ka Man So, Naresh Singh, Jia Wang, George E. Sandusky, Yunlong Liu, Mateusz Opyrchal, Sha Cao, Jun Wan, Chi Zhang, Xinna Zhang

**Affiliations:** ^1^ Department of Medical and Molecular Genetics Indiana University School of Medicine Indianapolis IN 46202 USA; ^2^ Melvin and Bren Simon Comprehensive Cancer Center Indiana University School of Medicine Indianapolis IN 46202 USA; ^3^ Center for Computational Biology and Bioinformatics Indiana University School of Medicine Indianapolis IN 46202 USA; ^4^ Department of Computer Science Indiana University Bloomington IN 47405 USA; ^5^ Division of Hematology/Oncology, Department of Medicine Indiana University School of Medicine Indianapolis IN 46202 USA; ^6^ Department of Pathology and Laboratory Medicine Indiana University School of Medicine Indianapolis IN 46202 USA; ^7^ Department of Biostatistics and Health Data Science Indiana University School of Medicine Indianapolis IN 46202 USA; ^8^ Department of Biomedical Engineering and Knight Cancer Institute Oregon Health & Science University Portland OR 97239 USA

**Keywords:** colorectal cancer, glutamine metabolism, immune checkpoint blockade, immunoproteasome, MHC‐I antigen presentation, single‐cell flux estimation analysis

## Abstract

Colorectal cancer (CRC) cells display remarkable adaptability, orchestrating metabolic changes that confer growth advantages, pro‐tumor microenvironment, and therapeutic resistance. One such metabolic change occurs in glutamine metabolism. Colorectal tumors with high glutaminase (GLS) expression exhibited reduced T cell infiltration and cytotoxicity, leading to poor clinical outcomes. However, depletion of GLS in CRC cells has minimal effect on tumor growth in immunocompromised mice. By contrast, remarkable inhibition of tumor growth is observed in immunocompetent mice when GLS is knocked down. It is found that GLS knockdown in CRC cells enhanced the cytotoxicity of tumor‐specific T cells. Furthermore, the single‐cell flux estimation analysis (scFEA) of glutamine metabolism revealed that glutamate‐to‐glutathione (Glu‐GSH) flux, downstream of GLS, rather than Glu‐to‐2‐oxoglutarate flux plays a key role in regulating the immune response of CRC cells in the tumor. Mechanistically, inhibition of the Glu‐GSH flux activated reactive oxygen species (ROS)‐related signaling pathways in tumor cells, thereby increasing the tumor immunogenicity by promoting the activity of the immunoproteasome. The combinatorial therapy of Glu‐GSH flux inhibitor and anti‐PD‐1 antibody exhibited a superior tumor growth inhibitory effect compared to either monotherapy. Taken together, the study provides the first evidence pointing to Glu‐GSH flux as a potential therapeutic target for CRC immunotherapy.

## Graphical abstract

Knockdown of GLS in CRC cells enhanced the cytotoxicity of tumor‐infiltrated CD8^+^ T cells. This study further demonstrated that glutamate‐to‐glutathione (Glu‐GSH) flux, downstream of GLS, plays a key role in regulating antitumor immunity. Mechanistically, inhibition of the Glu‐GSH flux activated ROS‐related signaling pathways in tumor cells, thereby increasing the tumor immunogenicity by promoting the activity of the immunoproteasome.

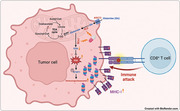

## 1 Introduction

Colorectal cancer (CRC) ranks as the second most common cancer in women and the third most common in men worldwide. It stands as the second‐leading cause of cancer‐related deaths overall and is the primary cause in men younger than 50 years.^[^ ^1^ ^]^ First‐line therapy of metastatic colorectal cancer relies on a combination of chemotherapy and targeted therapies, according to patient clinical characteristics and tumor molecular profile.^[^ ^2^ ^]^ Recent advances in immune checkpoint blockade (ICB) therapy have changed the course of cancer treatment. However, only a small fraction (approximately 5%) of CRC cases with a microsatellite instability‐high (MSI‐H) and/or mismatch repair deficiency (dMMR) phenotype are eligible for the current ICB therapy.^[^ ^3^ ^]^ Moreover, the complete response rate even in patients with MSI‐H/dMMR cancers was poor based on the results of multiple clinical trials.^[^ ^4^ ^]^ Therefore, there is an urgent need to identify interventions that improve the efficacy of ICB therapy for CRC patients.

In tumor immunology, CD8^+^ cytotoxic T cells recognize the tumor antigen bound to the major histocompatibility complex class‐I (MHC‐I) molecules on the surface of tumor cells, thereby selectively killing these tumor cells. Tumor antigens are degraded by the immunoproteasomes, and then degraded peptides are transported and processed by the precise coordination of several different subunits such as Transporter associated with Antigen Processing (TAP), peptide‐loading complex (PLC) and MHC‐I molecules.^[^ ^5^ ^]^ However, to evade T cell killing, cancer cells have developed mechanisms to suppress their tumor antigen presentation, including alterations in the MHC molecules or the antigen‐presenting machinery such as immunoproteasome subunits, TAP, and PLC.^[^ ^6^ ^]^ Impaired MHC‐I antigen processing and presentation have been proposed as an important mechanism of acquired resistance to ICB therapy in different types of cancer.^[^ ^7^ ^]^ Therefore, restoring antigen presentation of tumor cells is a promising strategy for boosting T cell‐mediated anti‐tumor responses.^[^ ^8^ ^]^

Cancer cells continually evolve and orchestrate cellular changes, resulting in the selection of the most advantageous clones.^[^ ^9^ ^]^ Reprogramming of metabolic pathways grants cancer cells superior capacities to proliferate, survive, and suppress anti‐tumor immunity.^[^ ^10^ ^]^ The heightened metabolic rate seen in rapidly dividing cancer cells not only impedes immune cell infiltration into tumors but also competes for resources within the tumor microenvironment (TME), thereby dampening the anti‐tumor capabilities of immune cells.^[^ ^11^ ^]^ As a result, therapeutic approaches targeting the metabolic reliance of colorectal cancer cells have the potential to rejuvenate T cell cytotoxicity and bolster anti‐tumor responses within the TME.^[^ ^10a^ ^]^

In rapidly growing cells, especially cancer cells, glutamine is avidly consumed for their energy production, and to provide carbon and nitrogen as building blocks for biomass accumulation.^[10b,^ ^12^ ^]^ Within the mitochondria, glutaminase (GLS) catalyzes the initial step of glutamine metabolism, converting glutamine to glutamate. Glutamate, in turn, is further converted to 2‐oxoglutarate (2‐OG, also known as α‐ketoglutarate) by glutamate dehydrogenase 1 (GLUD1) that enters the citric acid cycle (TCA cycle) or is metabolized to glutathione (GSH) by glutamate‐cysteine ligase catalytic subunit (GCLC) for maintaining cellular redox homeostasis.^[^ ^10^, ^13^ ^]^ Cancer cells manage to elevate GLS expression or enhance glutamine flux to fuel the synthesis of amino acids, nucleotides, or fatty acids. These glutamine‐dependent cancer cells rely heavily on glutamine and exhibit sensitivity to either glutamine deprivation or the inhibition of glutaminase.^[^ ^14^ ^]^ Given the essential role of glutamine metabolism in cancer development and growth,^[^ ^15^ ^]^ numerous glutaminolysis inhibitors have been developed and are being tested in clinical trials.^[^ ^16^ ^]^

In this study, we identified GLS as one of the top‐ranked metabolic genes that are most negatively correlated with T cell cytotoxicity levels in clinical datasets of human CRC. Inhibition of GLS in CRC cells promoted the antitumor immune response. Interestingly, we found that inhibition of GLS in tumor cells enhanced immunoproteasome gene expression, thereby activating the immunoproteasome and MHC‐I‐mediated tumor antigen presentation. Our in silico metabolic flux analysis revealed that the glutamate‐to‐glutathione (Glu‐GSH) flux played a major role in regulating T cell cytotoxicity. Furthermore, inhibition of GLS induced the level of reactive oxygen species (ROS) in tumor cells and activated the JAK/STAT1 pathway that upregulates the expression of immunoproteasome genes. Pharmacological inhibition of GCLC, a key enzyme in the Glu‐GSH flux, also elevated tumor antigen presentation and sensitized colorectal tumors to anti‐PD‐1 therapy. Our findings highlight the important role of glutamine metabolic flux in modulating tumor immune response.

## 2 Results

### 2.1 Inhibition of Glutamine Metabolism in CRC Cells Promotes Anti‐Tumor Immunity

**Figure 1:**
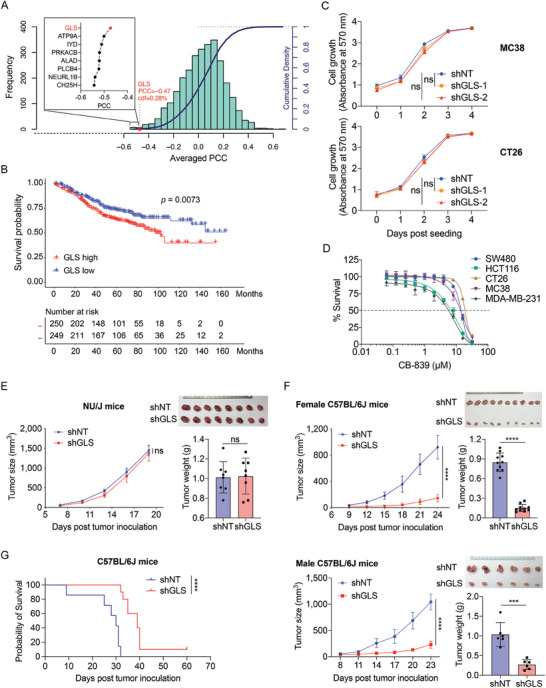
Inhibition of GLS in tumor cells suppresses tumor growth in immunocompetent mice. A) The average Pearson correlation coefficient (PCC) between metabolic genes and T cell cytotoxicity from the nine CRC datasets was analyzed using the Inference of Cell Types and Deconvolution (ICTD). The histogram and the curve show the distribution and cumulative distribution function (cdf) of the PCC, respectively. The average PCC between GLS and T cell cytotoxicity was highlighted. The top 8 enzyme genes that were negatively associated with the relative cytotoxicity of T cells and their average PCC were shown. B) Kaplan–Meier survival curve from the GSE39582 (n = 505) dataset of CRC cases with high and low GLS expression (top and bottom 40%). C) Cell proliferation of MC38 and CT26 cell lines expressing control (shNT) or GLS shRNA (shGLS). The cell proliferation was determined using violet crystal staining at indicated time points. Data were presented as mean ± SD (n = 5) and analyzed using One‐way ANOVA and Dunnett's multiple comparisons test for the absorbance at the endpoint. D) Inhibition curves of CRC cell lines upon CB‐839 treatment, the dashed line indicates 50% survival of the cells. E,F) MC38 cells with control or GLS knockdown were inoculated subcutaneously into female NU/J mice (E), and female and male C57BL/6J mice (F). The tumor images and tumor weights were taken at the endpoint. The tumor sizes were measured at indicated time points. Data were analyzed using the unpaired two‐tailed *t*‐test for the tumor sizes and weights at the endpoint and presented as mean ± SD (E, n = 8; F, female mice n = 10; F, male mice n = 6). G) The overall survival of C57BL/6J mice orthotopically implanted with MC38 cells expressing shNT and shGLS. Data were analyzed using Log‐rank (Mantel–Cox) test, shNT, n = 7; shGLS, n = 10.

The heightened demand for nutrients, metabolites, and oxygen by rapidly proliferating cancer cells, along with the immunosuppressive by‐products they generate, results in challenging environmental conditions for immune cells to navigate and function.^[^ ^17^ ^]^ To understand metabolic events in cancer cells that contribute to the establishment of an immunosuppressive TME, we conducted an analysis of The Cancer Genome Atlas (TCGA) and eight other high‐quality CRC transcriptomics datasets using the Inference of Cell Types and Deconvolution (ICTD) algorithm.^[^ ^18^ ^]^ We computed and ranked metabolic genes whose expression levels consistently exhibited a negative correlation with T cell cytotoxicity in nine CRC datasets (**Figure** **1**A). Among the top eight genes, GLS is of particular interest due to its role in glutaminolysis, a hallmark of reprogrammed metabolism in cancer cells and a potential target for cancer therapy.^[^ ^13^, ^16^ ^]^ To determine the clinical relevance of GLS, we analyzed the GSE39582 (n = 505) dataset. Decreased overall survival was observed in the patients with GLS‐high expression, as compared with the GLS‐low group (Figure 1B), and the 5‐year cutoff survival curve is shown in Figure S1A (Supporting Information).

It was reported that the PIK3CA‐mutant CRC cell lines are sensitive to glutamine deprivation.^[^ ^19^ ^]^ However, the knockdown of GLS (GLS‐KD) did not significantly affect the proliferation of both PIK3CA‐wildtype (PIK3CA‐WT, MC38, CT26, and SW480) and PIK3CA‐mutant (HCT116) cells (Figure 1C; Figure S1B, Supporting Information). We also examined the effect of GLS inhibition (using GLS inhibitor CB‐839)^[^ ^20^ ^]^ on the proliferation of these cells. The MDA‐MB‐231 is a PIK3CA‐mutant human breast cancer cell line that was reported to be sensitive to glutamine restriction.^[^ ^21^ ^]^ Consistent with the result of GLS knockdown study, the PIK3CA‐mutant cell lines were not remarkably more sensitive to CB‐839 treatment than the wildtype cell lines, with growth inhibitory 50% (GI_50_) of 8.03 µM (HCT116), 7.93 µM (MDA‐MB‐231), 15.78 µM (MC38), 16.25 µM (CT26), and 15.76 µM (SW480), respectively (Figure 1D). Our analysis also revealed that patients with high GLS expression tended to exhibit poorer survival rates compared to those with low GLS expression (Figure S1C, Supporting Information), both in PIK3CA WT and mutant groups, and GLS expression was negatively correlated with CD8^+^ T cell infiltration in both PIK3CA WT and mutant tumors, suggesting that the correlation existed regardless of PIK3CA status (Figure S1D, Supporting Information). The in vitro results were also validated in vivo by inoculating MC38 cells into the immunocompromised NU/J mice, where tumor growth was similar between control and GLS‐KD groups (Figure 1E). Surprisingly, a significant delay in the growth of the GLS‐KD tumors was observed in immunocompetent mice bearing MC38 (C57BL/6) and CT26 (BALB/c) cells‐derived tumors in both genders (Figure 1F; Figure S1E,F, Supporting Information). We further inoculated the MC38 cells orthotopically into the cecal wall of C57BL/6 mice.^[^ ^22^ ^]^ Consistently, mice in the GLS‐KD group lived significantly longer than mice in the control group (Figure 1G). Those findings suggest that interfering with glutamine metabolism by targeting GLS potentially promotes anti‐tumor immunity.

### 2.2 Inhibition of GLS in Tumor Cells Enhances their Immune Response to CD8^+^ T Cells

**Figure 2:**
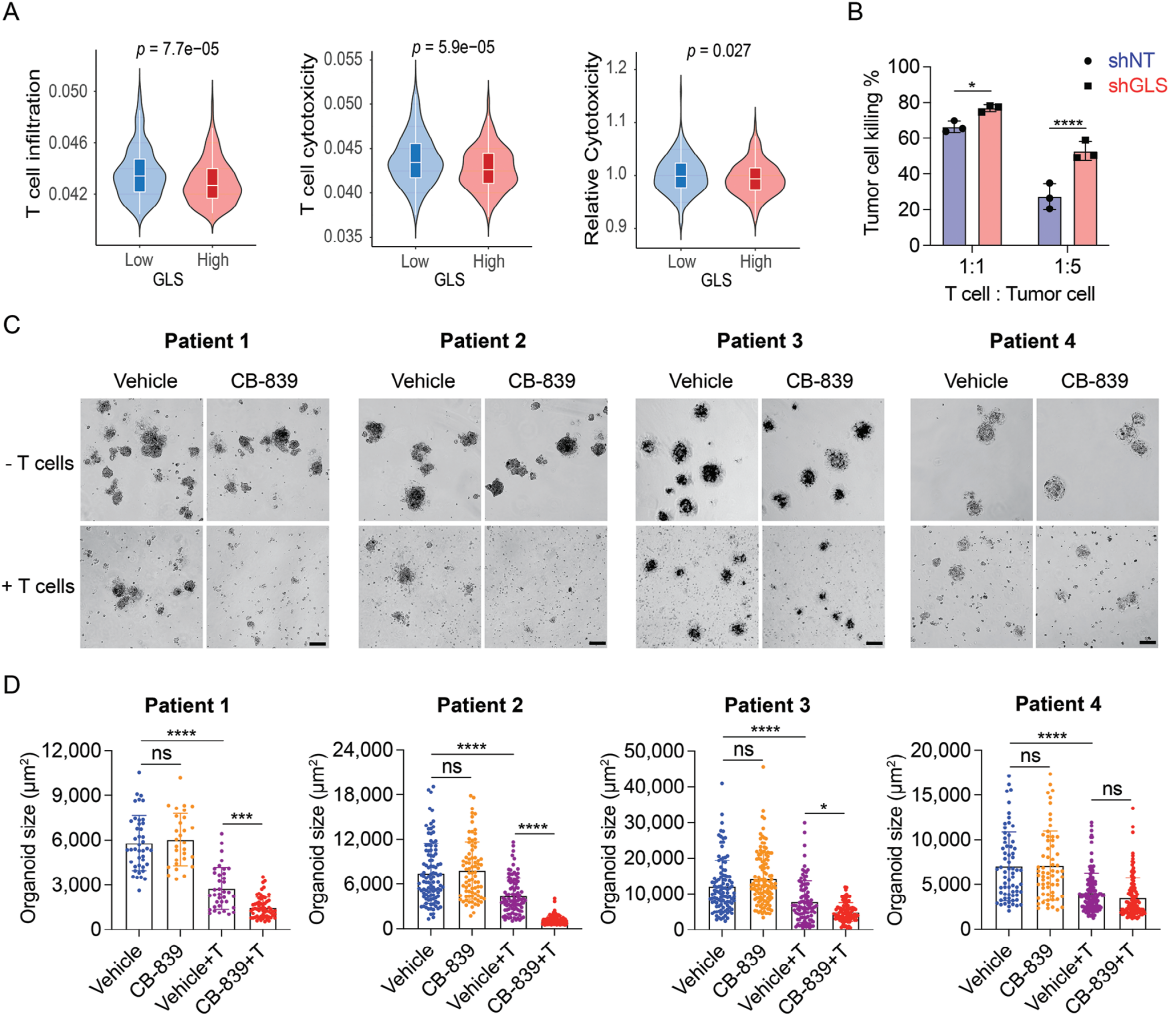
Inhibition of GLS in tumor cells enhances CD8^+^ T cell cytotoxicity. A) The Violin Plots represent the tumor infiltration, cytotoxicity, and relative cytotoxicity level of CD8^+^ T cells in human CRC cases with high or low GLS expression levels. The GSE39582 dataset was used for the analysis. The unpaired two‐tailed *t*‐test was used for statistical analysis. B) CD8^+^ T cell cytotoxicity assay. CD8^+^ T cells were isolated from OT‐I mouse, activated with mouse CD3/CD28 beads and murine IL‐2, and then co‐cultured with MC38 cells that expressed control/GLS shRNA and luciferase and were preloaded with SIINFEKL (OVA) peptides. The tumor cell killing was measured 16 h post‐co‐culture using luciferase assay. Data were analyzed using the unpaired two‐tailed *t*‐test and presented as mean ± SD (n = 3). C,D) Patient‐derived organoid killing assay. Autologous CD8^+^ T cells were isolated from the patient tissue and activated in vitro with human CD3/CD28 beads and IL‐2. The organoids were treated with CB‐839 at 2 µM for 2 days and then co‐cultured with or without the CD8^+^ T cells for 24 h. Representative images (scale bar = 100 µm) of the organoids were shown in (C), and the sizes of the organoids were quantified using Image J (D). Data were presented as mean ± SD. Data were analyzed using ordinary one‐way ANOVA and Tukey's multiple comparisons test (*n* = 30–100 organoids).

We further analyzed bulk RNA‐seq datasets of human colorectal tumors to evaluate the correlation between T cell infiltration and cytotoxicity with GLS expression levels. The results showed that high GLS expression was associated with decreased T cell infiltration and dampened effector functions (**Figures** **2**A; S2A, Supporting Information). The CRC cells have a higher level of GLS expression in comparison with stromal and immune cells in the TME by analyzing cell line data (Figure S2B, Supporting Information). Our analysis suggests that high levels of GLS expression in the cancer cells may impact the cytotoxicity of CD8^+^ T cells in the tumor. To determine it, we first isolated CD8^+^ T cells from OT‐I transgenic mice^[^ ^23^ ^]^ and co‐cultured them with MC38 cells expressing control shRNA (shNT) or *Gls* shRNA (shGLS). The MC38 cells were pre‐loaded with OVA_257–264_ peptides. Knockdown of GLS in MC38 cells remarkably enhanced the OT‐I T cell cytotoxicity (Figure 2B). Next, we co‐cultured patient‐derived organoids (PDOs) with autologous T cells isolated and amplified from the same patient tumor tissue. The GLS inhibitor CB‐839 was used to pretreat the organoids before co‐culturing. CB‐839 treatment had no effect on the growth of tumor organoids, but remarkably potentiated autologous CD8^+^ T cytotoxicity (determined by the quantification of organoid sizes) in 3 (Patient #1–3) out of 4 patient tumor tissues (Figure 2C,D; Table S1, Supporting Information). The results indicate that inhibiting GLS in tumor cells enhances their sensitivity to CD8^+^ T cell killing.

### 2.3 Depletion of GLS in CRC Cells Alters the Tumor Immune Microenvironment

**Figure 3:**
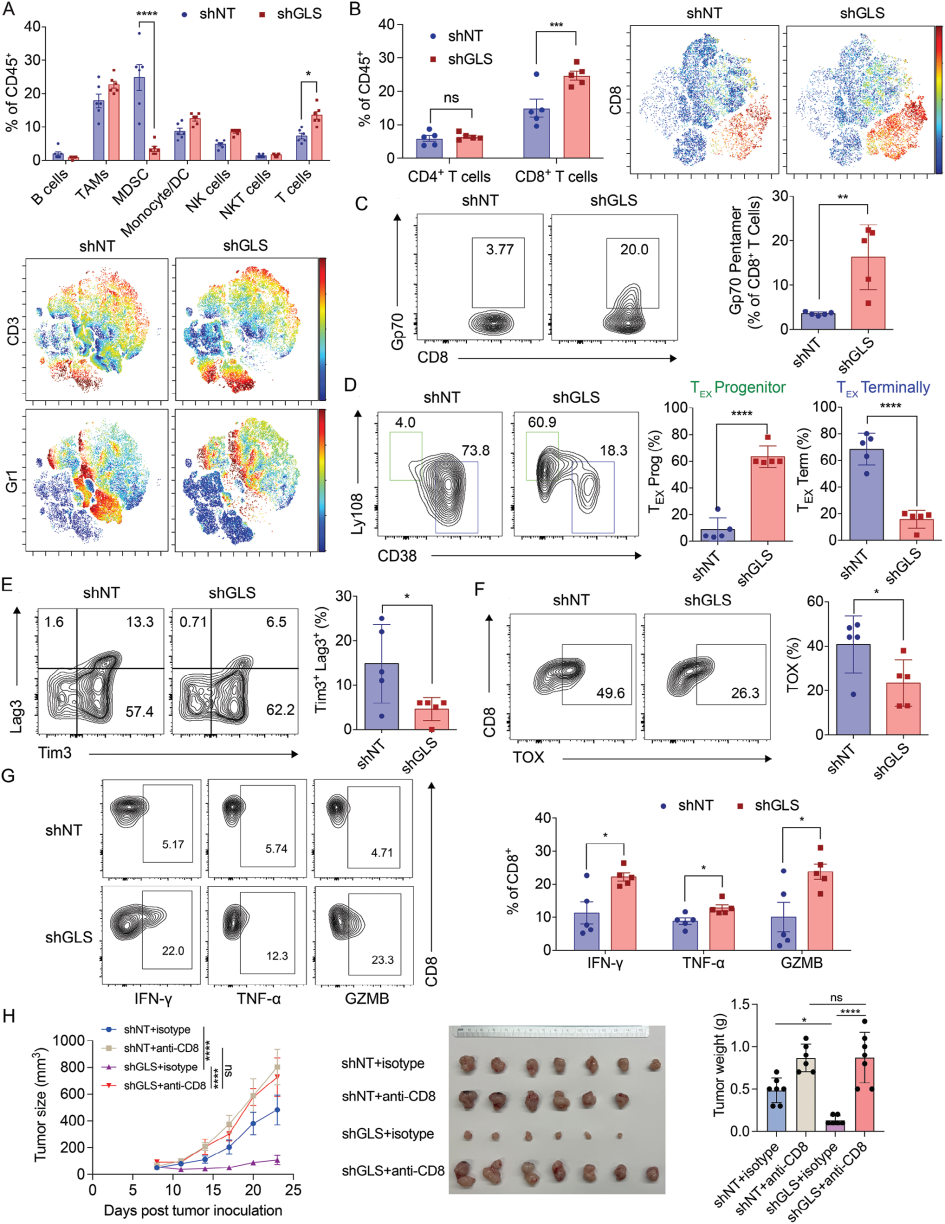
Inhibition of GLS in tumor cells potentiates the CD8^+^ T cell‐mediated immune responses in mice. A) Flow cytometry analysis of tumor microenvironment changes in MC38 control and GLS‐KD tumors. Data were analyzed using two‐way ANOVA and Sidak's multiple comparisons test (n = 6; presented as mean ± SEM). Representative results of t‐distributed stochastic neighbor embedding (t‐SNE) representation of CD3 and Gr1 positive cells in control and GLS‐KD tumors are shown at the bottom. B) The percentages of CD4^+^ and CD8^+^ T cells in the abovementioned tumors were analyzed using two‐way ANOVA and Sidak's multiple comparisons test (n = 5; presented as mean ± SEM). Representative results of t‐SNE representation of CD8 positive cells in control and GLS‐KD tumors are shown on the right. C) Flow cytometry analysis of the tumor‐specific CD8^+^ T cells. Representative contour plots of Gp70 on CD8^+^ T cells were shown (left) and the data were analyzed (right) using the unpaired two‐tailed *t*‐test (n = 5; data displayed as mean ± SD). D) Representative contour plots of Ly108 and CD38 markers on CD8^+^ T cells with quantification. T_EX_, exhausted T cells. Data were analyzed using the unpaired two‐tailed *t*‐test (n = 5; presented as mean ± SD). E) Representative contour plots of Lag3 and Tim3 markers on CD8^+^ T cells with quantification. Data were analyzed using the unpaired two‐tailed *t*‐test (n = 5; presented as mean ± SD). F) Representative contour plots of TOX on CD8^+^ T cells with quantification. Data were analyzed using the unpaired two‐tailed t‐test (n = 5; presented as mean ± SD). G) CD8^+^ T cells isolated from MC38‐derived control and GLS‐KD tumors were analyzed by flow cytometry for their activity indicated by interferon‐gamma (IFN‐γ), tumor necrosis factor‐alpha (TNF‐α), and granzyme B (GZMB) levels in the cells. Data were analyzed using the unpaired two‐tailed t‐test (n = 5; presented as mean ± SEM). H) MC38 cells with control or GLS knockdown were inoculated subcutaneously into female C57BL/6J mice. The isotype and anti‐CD8 antibodies were administrated two days before the tumor cell inoculation and three times a week throughout the experiment. The tumor sizes were measured at indicated time points and data were analyzed using two‐way ANOVA and Turkey's multiple comparisons test. The tumor images and tumor weights were taken at the endpoint and data were analyzed using one‐way ANOVA and Tukey's multiple comparisons test. Data were presented as mean ± SD (n = 7 for shNT+isotype and shGLS+anti‐CD8; n = 6 for shNT+anti‐CD8 and shGLS+isotype).

Since significant tumor growth inhibition was observed in immunocompetent mice with GLS depletion, we wanted to determine which tumor‐infiltrating lymphocytes were affected by the change of GLS levels in tumors. Therefore, we inoculated the control and GLS‐KD MC38 cells into C57BL/6 mice and assessed their tumor immune cell profiles using flow cytometry (Figure S3A,B, Supporting Information). Our data revealed mainly a significant decrease in myeloid‐derived suppressor cells (MDSC, CD11b^+^/Gr1^+^) and an increase in T cells in GLS‐KD tumors (**Figure** **3**A). The infiltration of other cell types such as B cells, nature killer (NK) cells, NKT cells, tumor‐associated macrophages (TAMs), and monocytes/dendritic cells (DCs) were not significantly different between the control and GLS‐KD tumors (Figure 3A). Furthermore, the myeloid cell types including MDSCs, TAMs, and monocytes/DCs displayed enhanced levels of MHC‐II in the GLS‐KD tumors, suggesting an elevation of antigen‐presentation function (Figure S3C, Supporting Information). Using an orthotopic model (MC38 cells injected into the cecal wall of C57BL/6 mice), we collected and analyzed the immune microenvironment in the shNT and GLS KD tumors. The results were consistent with those of the subcutaneous model, showing an upregulation of the CD8^+^ T cell population in the GLS inhibition group compared to the controls. However, unlike in the subcutaneous model, we did not observe a change in the MDSC population upon GLS inhibition, which may reflect differences in the immune microenvironments between the two models (Figure S4A, Supporting Information).

When looking more specifically into the T cell subpopulations of GLS‐KD tumors, we found that only CD8^+^ T cells were increased while no significant difference was found for CD4^+^ T cells (Figure 3B). However, given the diverse functions and responses of different CD4^+^ T cell subtypes in cancer surveillance, and the requirement for additional conditions to activate CD4^+^ T cells, it's possible that some subpopulations of CD4^+^ T cells may be upregulated, but this is not yet well understood. Gp70 is a well‐known antigen expressed by MC38 tumor cells.^[^ ^24^ ^]^ Using Gp70 pentamers, we were able to detect MC38‐specific CD8^+^ T cells in the tumors. The GLS‐KD tumors were infiltrated to a much higher extent with the Gp70‐positive CD8^+^ T cells compared to the control tumors, in support of a tumor‐specific immune response (Figure 3C). Many aspects such as specificity, activation, and exhaustion can determine the pivotal role of CD8^+^ T cells in driving antitumor immunity.^[^ ^25^ ^]^ To address whether those tumor‐infiltrating CD8^+^ T cells are functional, we determined their activation and exhaustion status. Despite the existence of multiple subtypes of exhausted T cells in tumors,^[^ ^26^ ^]^ we were interested in two subpopulations: exhausted progenitor (T_EX_ progenitor) and terminally exhausted (T_EX_ terminally). Particularly, a subset of CD8^+^ T cells (T_EX_ progenitor) with stem cell‐like features respond well to anti‐PD1 checkpoint inhibition.^[^ ^27^ ^]^ Based on the markers Ly108 and CD38, we observed an increased number of T_EX_ progenitor cells in the GLS‐KD tumors as compared to the control tumors (Figure 3D). This was also confirmed by the fact that the CD8^+^ T cells in the GLS‐KD tumors displayed a reduced level of CD39^+^, which is often associated with T cell exhaustion (Figure S3D, Supporting Information). Interestingly, the CD8^+^ T cells in the GLS‐KD tumors had an increased level of CD103 expression (Figure S3D, Supporting Information). This specific subset of CD103^+^ T cells was also found to be correlated to improved patient outcomes.^[^ ^28^ ^]^ In addition, we observed a reduced number of CD8^+^ T cells expressing high levels of inhibitory markers including Lag3 and Tim3 in the GLS‐KD tumors (Figure 3E). Furthermore, the depletion of GLS significantly lowered the frequency of exhausted TOX‐expressing CD8^+^ T cells (Figure 3F).^[^ ^29^ ^]^ Importantly, CD8^+^ T cells in the GLS‐KD tumors exhibited enhanced levels of cytotoxicity markers (IFN‐γ, TNF‐α, and Granzyme B) (Figure 3G; Figure S3E,F, Supporting Information). The depletion of CD8^+^ T cells abolished the tumor‐inhibiting effects of GLS knockdown, demonstrating that CD8^+^ T cells play a crucial role in mediating antitumor immunity following GLS inhibition (Figure 3H; Figure S4B, Supporting Information). Taken together, GLS‐KD tumors had enhanced levels of tumor‐specific effective CD8^+^ T cells for eliciting an antitumor response.

### 2.4 Inhibition of GLS in CRC Cells Enhances the Activity of Immunoproteasome

**Figure 4:**
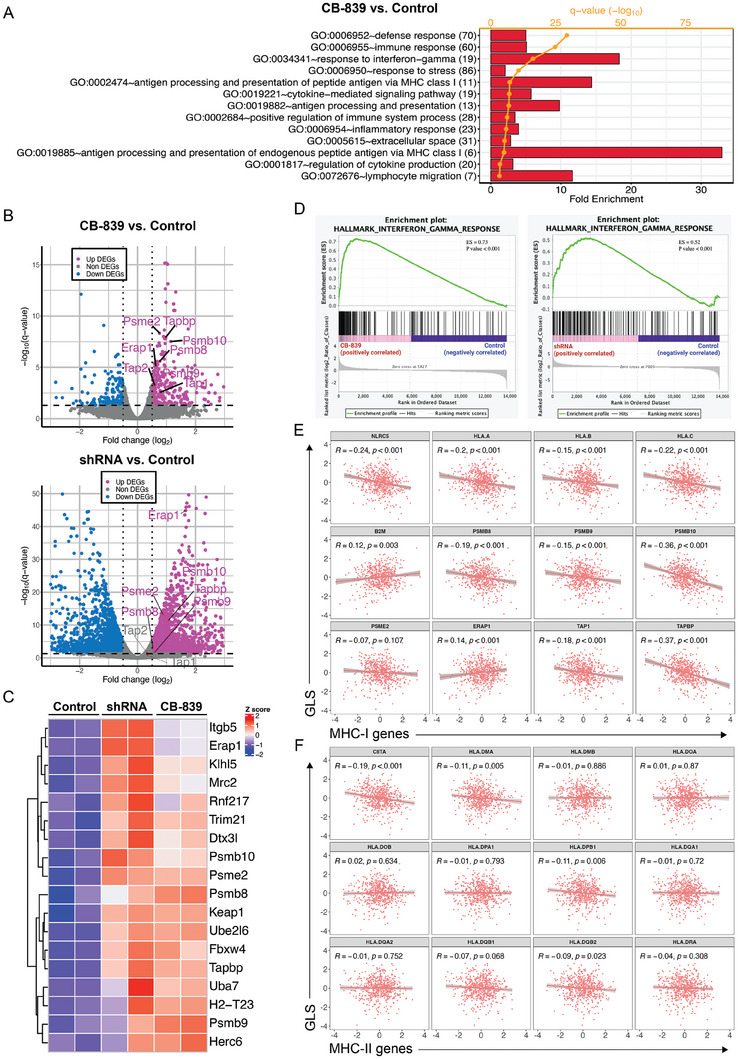
Inhibition of GLS in tumor cells enhances the expression of immunoproteasome genes. A) Gene ontology pathway analysis of mRNA‐seq data for control and CB‐839 treated MC38 cells. B) Volcano plot analysis of mRNA‐seq data from CB‐839 versus the control group and shGLS versus the control group. Genes of interest (immunoproteasome genes) were labeled. C) Heatmap of expression of genes that were involved in antigen presentation pathway. D) Gene signature analysis for IFN‐γ responses of mRNA‐seq data from CB‐839 versus control and shGLS versus control groups. E) Correlation analysis of *GLS* and MHC‐I‐mediated antigen presentation genes using the Colorectal Adenocarcinoma (TCGA, PanCancer Atlas) dataset. F) Correlation analysis of *GLS* and MHC‐II‐mediated antigen presentation genes using the Colorectal Adenocarcinoma (TCGA, PanCancer Atlas) dataset.

To study the mechanism by which inhibiting glutamine metabolism sensitizes tumor cells to T cell killing, we performed mRNA sequencing (mRNA‐seq) on MC38 cells with GLS inhibition (CB‐839) or knockdown (shRNA). The gene ontology (GO) enrichment analysis showed a number of significantly changed pathways that potentially contribute to the enhanced T cell cytotoxicity upon GLS inhibition (**Figure** **4**A). Particularly, the GO pathways (GO: 0 019 885, 0 002 474, and 0 019 882), related to MHC class I mediated antigen processing and presentation, stood out with the highest fold enrichment (Figure 4A). The differentially expressed genes in the MHC‐I antigen presentation pathway include *Erap1*, *Tapbp*, *Tap1*, *Tap2*, *Psme2*, *Psmb8*, *Psmb9*, and *Psmb10*. These genes were significantly upregulated in the GLS inhibitor treatment group as compared to the control group, and most of them (except *Tap1* and *Tap2*) were also upregulated in the GLS‐KD group (Figure 4B). As many as 18 genes associated with the MHC‐I‐mediated antigen processing and presentation (according to the PathCards pathway unification database) were upregulated upon GLS inhibition or knockdown (CB‐839 versus Control and GLS‐KD versus Control) (Figure 4C). Our pathway analysis also revealed that the response to the interferon‐γ (IFN‐γ) pathway (GO: 0 034 341) was enhanced upon GLS inhibition, which is consistent with the previous report that IFN‐γ drives the expression of genes in MHC‐I antigen processing and presentation via activation of JAK/STAT1 signaling.^[^ ^30^ ^]^ We then applied the Gene Set Enrichment Analysis (GSEA)^[^ ^31^ ^]^ and Molecular Signature Database (MSigDB)^[^ ^32^ ^]^ and identified that the IFN‐γ Response Gene Set was enriched in the CB‐839 treatment or GLS‐KD group as compared to the control group (Figure 4D). We further analyzed the co‐expressions between key genes in MHC‐I or MHC‐II antigen presentation and *GLS* using the TCGA colorectal adenocarcinoma dataset. The expression levels of immunoproteasome genes, as well as other key genes that are involved in MHC‐I antigen presentation, were negatively correlated with the expression of *GLS* (Figure 4E). However, no significant correlation between MHC‐II genes with *GLS* was found (Figure 4F). Taken together, the results suggest a regulatory role of glutamine metabolism in the MHC‐I antigen presentation pathway.

**Figure 5:**
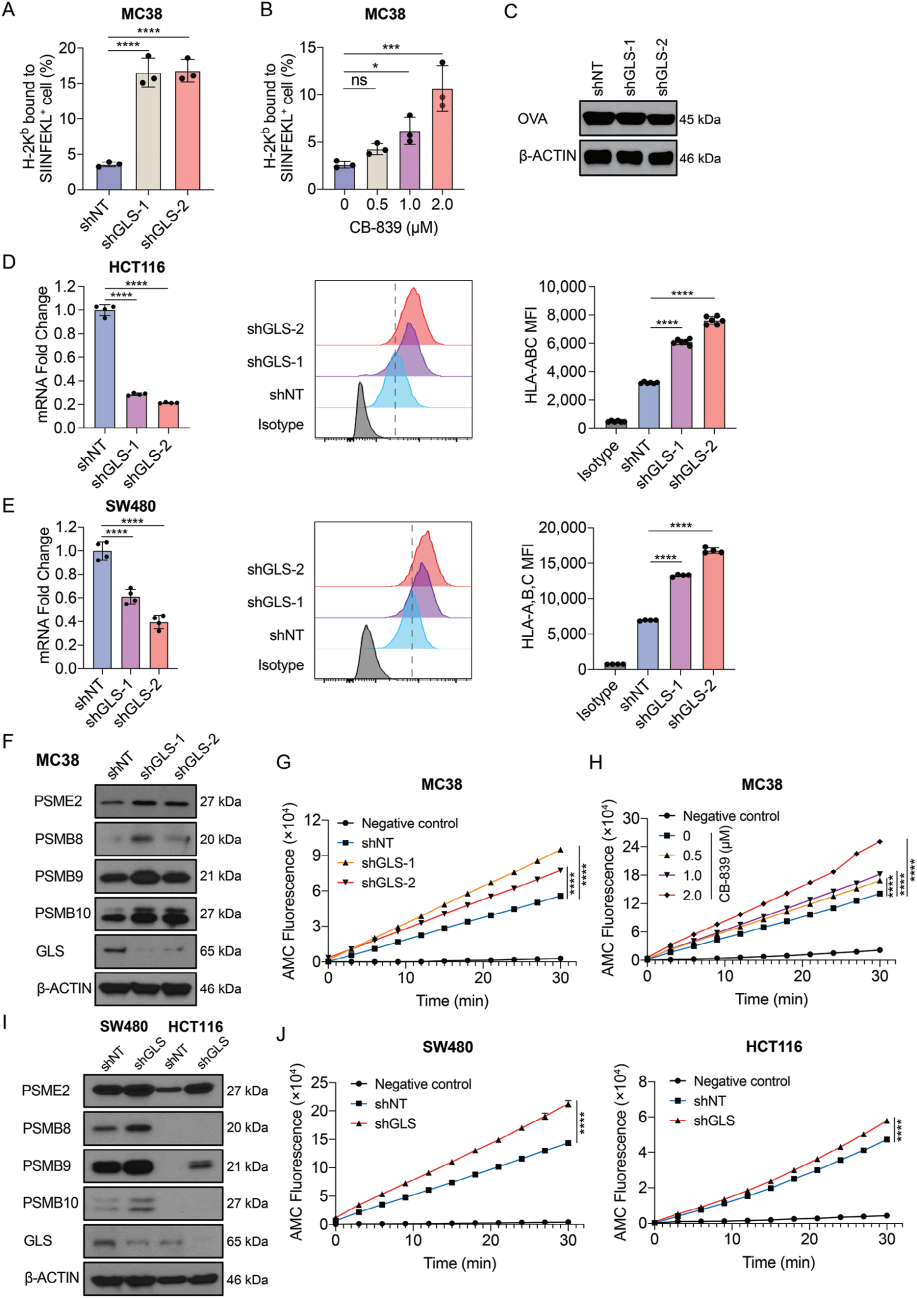
Inhibition of GLS activates immunoproteasome and enhances tumor antigen presentation. A) H‐2K^b^ mediated SIINFEKL (OVA) presentation in control or GLS‐KD MC38 cells was determined by flow cytometry. Data were analyzed using One‐way ANOVA and Dunnett's multiple comparisons test and presented as mean ± SD (n = 3). B) H‐2K^b^ mediated SIINFEKL (OVA) presentation in control or MC38‐OVA cells treated with CB‐839 at the indicated doses was determined by flow cytometry. Data were analyzed using one‐way ANOVA and Dunnett's multiple comparisons test and presented as mean ± SD (n = 3). C) Ovalbumin (OVA) overexpression levels in MC38 cells with control or GLS KD were determined by Western blotting, and β‐ACTIN was used as a loading control. D,E) *GLS* mRNA expression levels were determined using qPCR. Cell surface HLA‐A,B,C levels were determined using flow cytometry in control and shGLS expressing human CRC cell lines. Data were analyzed using One‐way ANOVA and Dunnett's multiple comparisons test and presented as mean ± SD (n = 6 for HCT116; n = 4 for SW480). F) Protein expression levels of immunoproteasome genes were determined by Western blotting in control and GLS‐KD MC38 cells G) Immunoproteasome activities of control and GLS‐KD MC38 cells were determined. Data were analyzed using the unpaired two‐tailed *t*‐test for the fluorescence at the endpoint and presented as mean ± SD (n = 3). H) Immunoproteasome activities were determined in control and CB‐839‐treated MC38 cells at the indicated doses. Data were analyzed using the unpaired two‐tailed *t*‐test for the fluorescence at the endpoint and presented as mean ± SD (n = 3). I) Protein expression levels of immunoproteasome genes were determined by Western blotting in control and GLS‐KD human CRC cells. J) Immunoproteasome activities were determined in control and GLS‐KD human CRC cells. Data were analyzed using the unpaired two‐tailed *t*‐test for the fluorescence at the endpoint and presented as mean ± SD (n = 3).

Among those upregulated genes upon GLS inhibition, there are genes mostly involved in the immunoproteasome formation (*Psme2*, *Psmb8*, *Psmb9*, and *Psmb10*). The 26S proteasome complex is in charge of the ubiquitin‐mediated protein degradation. When the three catalytic β subunits β1, β2, and β5 in the 26S proteasome are replaced by the inducible subunits β1i (PSMB9), β2i (PSMB10), and β5i (PSMB8), respectively, the immunoproteasome is formed.^[^ ^33^ ^]^ The specialized immunoproteasome with altered peptide cleavage properties is solely responsible for antigen processing and presentation on MHC‐I molecules.^[^ ^34^ ^]^ Low expression of immunoproteasome subunits in early‐stage non‐small cell lung carcinoma patients was associated with recurrence, metastasis, and a poor outcome,^[^ ^35^ ^]^ and high expression of immunoproteasome genes is associated with improved survival in breast cancer.^[^ ^36^ ^]^ Our mRNA‐seq results strongly indicate that suppressing GLS could potentially boost the formation of the immunoproteasome, leading to an enhanced MHC‐I antigen presentation, which in turn increases T cell cytotoxicity. To test this hypothesis, we expressed full‐length OVA in control and GLS‐KD MC38 cells. Using an antibody that detects H‐2K^b^ bound to SIINFEKL (OVA_257‐264_) peptide, we found that knockdown (**Figure** **5**A) or pharmacological inhibition (Figure 5B) of GLS in tumor cells enhanced MHC‐I‐mediated OVA antigen presentation and this increased presentation was not due to the alteration of intracellular OVA expression levels (Figure 5C). Similar results were observed in both PIK3CA‐WT and mutant human CRC cell lines with enhanced HLA‐A,B,C levels on the cell surface after knockdown of *GLS* (Figure 5D,E) or treatment with CB‐839 (Figure S5A,B, Supporting Information), suggesting that GLS inhibition‐induced tumor antigen presentation is independent of PIK3CA status. The *GLS* mRNA expression levels were similar in those cells (Figure S5C, Supporting Information). We next examined the expression of those immunoproteasome genes. Knockdown of GLS in both murine and human CRC cell lines upregulated protein levels of PSME2, PSMB8, PSMB9, and PSMB10 (Figure 5F,I). Consistently, the knockdown or pharmacological inhibition of GLS dramatically enhanced immunoproteasome activity in both murine and human CRC cell lines (Figure 5G,H,J). To determine whether the immunoproteasome activity determines the response to the CB‐839 treatment in the PDO experiment (Figure 2C,D), we examined the expression of these immunoproteasome genes in the PDOs treated with or without CB‐839. Interestingly, all the responders (Patient 1–3) in our study showed increased expression of immunoproteasome genes, while the expression of those genes remained unchanged in the cells of the non‐responder (Patient 4) upon CB‐839 treatment (Figure S5D, Supporting Information). In addition, the knockdown of GLS did not affect the expression of *H2‐k1*, a gene encodes for MHC class I molecule, in mouse MC38 cells (Figure S5E, Supporting Information). Together, these results demonstrate that inhibition of GLS activates the immunoproteasomes and enhances the MHC‐I antigen presentation in tumor cells.

### 2.5 The Glu‐GSH Flux Plays a Major Role in Regulating T Cell Cytotoxicity in Colorectal Tumors

**Figure 6:**
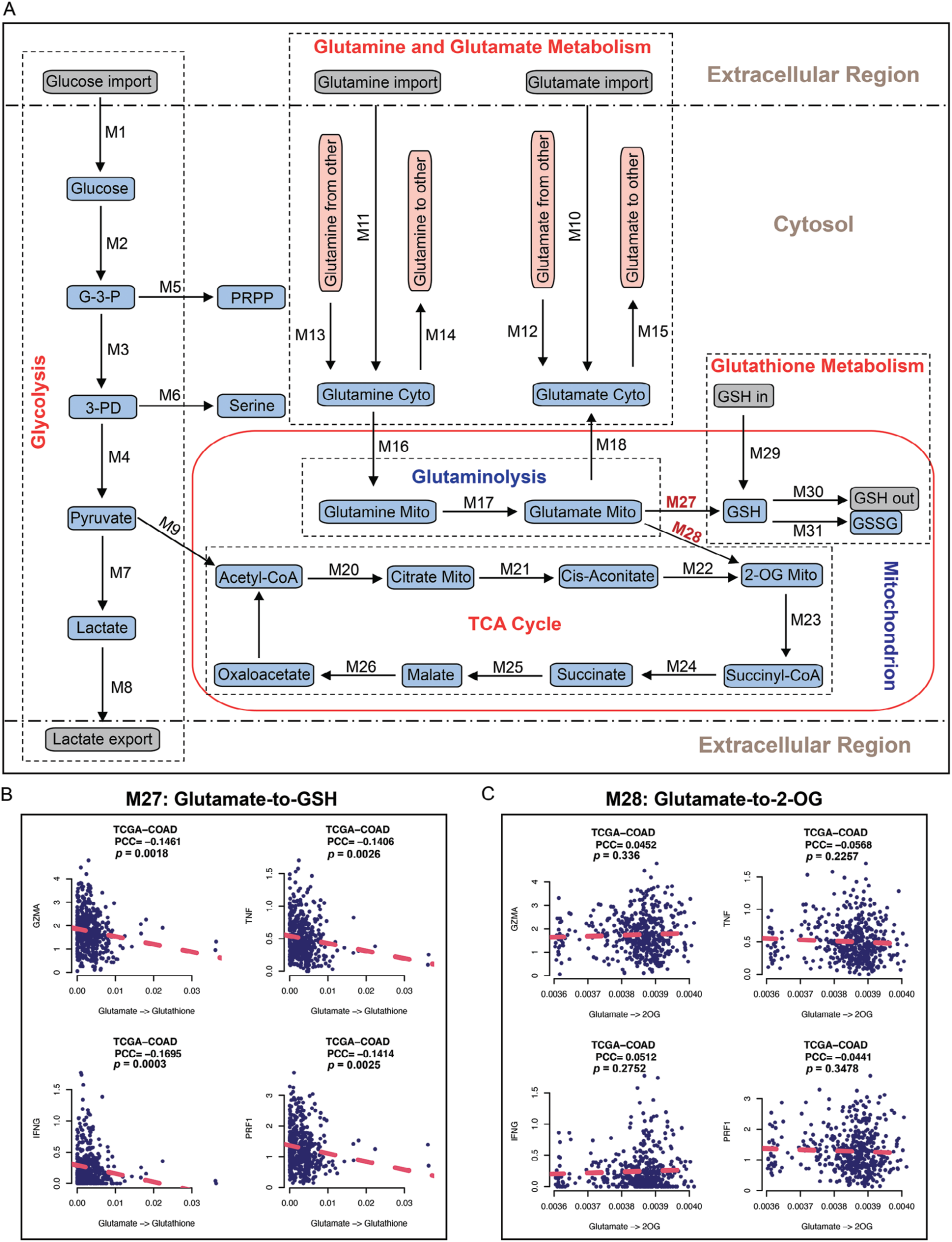
In silico metabolic flux analysis reveals the role of glutamate‐to‐GSH flux in regulating T cell cytotoxicity. A) Schematic diagram of the metabolic pathways, labeling with metabolic flux identifier numbers (Mx). B,C) The correlation between the metabolic flux analysis of glutamate‐to‐GSH (M27) (B), and glutamate‐to‐2‐OG (M28) (C) with the expression levels of CD8^+^ T cell effector marker genes.

Glutamine is the major amino acid avidly consumed by cancer cells to support their proliferation and survival. Once glutamine enters the cells through SLC1A5 (ASCT2), GLS converts it to glutamate in the mitochondria. Glutamate can then be metabolized either to GSH by GCLC or to 2‐OG by GLUD1 and then enter the TCA cycle.^[^ ^10b^ ^]^ To find out which metabolic pathway(s) downstream of GLS plays a primary role in regulating tumor antigen presentation, we performed in silico metabolic flux analysis using TCGA datasets. A computational method, named scFEA (Single‐cell Flux Estimation Analysis), was recently developed in our group to estimate the flux of the curated central metabolism network on the TCGA data.^[^ ^37^ ^]^ scFEA utilizes a graph neural network architecture to approximate the non‐linear dependency between the metabolic flux of each reaction module and the transcriptomic changes of genes involved in the module. Two computational assumptions were utilized by scFEA, including (i) the flux rate of each metabolic module can be modeled as a neural network of the genes involved in the module and (ii) the imbalance between the predicted in‐flux and out‐flux for intermediate metabolites should be minimized. The inputs of scFEA include transcriptomics data and a factor graph‐based representation of the metabolic map. The main output of scFEA is predicted sample‐wise metabolic flux, such as glutamine metabolic flux. We first reconstructed the metabolic pathways of glucose, glutamine, glutamate, and glutathione metabolism in a subcellular resolution (**Figure** **6**A). The reconstructed central metabolic network includes 31 reaction modules and 253 genes that cover the glycolysis, upstream and downstream of the TCA cycle, glutaminolysis, glutamine, glutamate, and glutathione metabolism, and three branches in the cytosol, mitochondrion, and extracellular region (see details in Experimental Section and Tables S2 and S3, Supporting Information). The glutamine metabolic flux of each reaction was estimated using scFEA and the correlations between the predicted flux of the downstream reactions of glutamate and the T cell cytotoxicity were further analyzed using the ICTD.^[^ ^18^ ^]^

Our metabolic flux analysis revealed that the Glu‐GSH flux (M27), but not the glutamate‐to‐2‐OG (Glu‐2‐OG) flux (M28), was negatively correlated with T cell effector genes, including *GZMA*, *TNF*, *IFNG*, and *PRF1* (Figure 6B,C). Consistent with the flux analyses, the high expression of GCLC, which catalyzed the synthesis of GSH in M27, was associated with low T cell cytotoxicity in multiple CRC datasets from TCGA (Figure S6B, Supporting Information). However, this type of negative correlation was not observed between GLUD1 expression and T cell cytotoxicity in the same datasets (Figure S6D, Supporting Information). Moreover, the epithelial cells have the highest level of GCLC expression in comparison to fibroblast and immune cells in the TME from the scRNA‐seq data of CRC samples (Figure S6A,C, Supporting Information). In sum, those findings highly suggest that the Glu‐GSH metabolic pathway uniquely regulates T cell cytotoxicity. GCLC is downstream of GLS in glutamine metabolism. Therefore, inhibiting GCLC could be a more specific therapeutic approach for CRC immunotherapy than inhibiting GLS.

### 2.6 Inhibition of Glu‐GSH Flux Enhances Tumor Antigen Presentation in a ROS‐Dependent Manner

**Figure 7:**
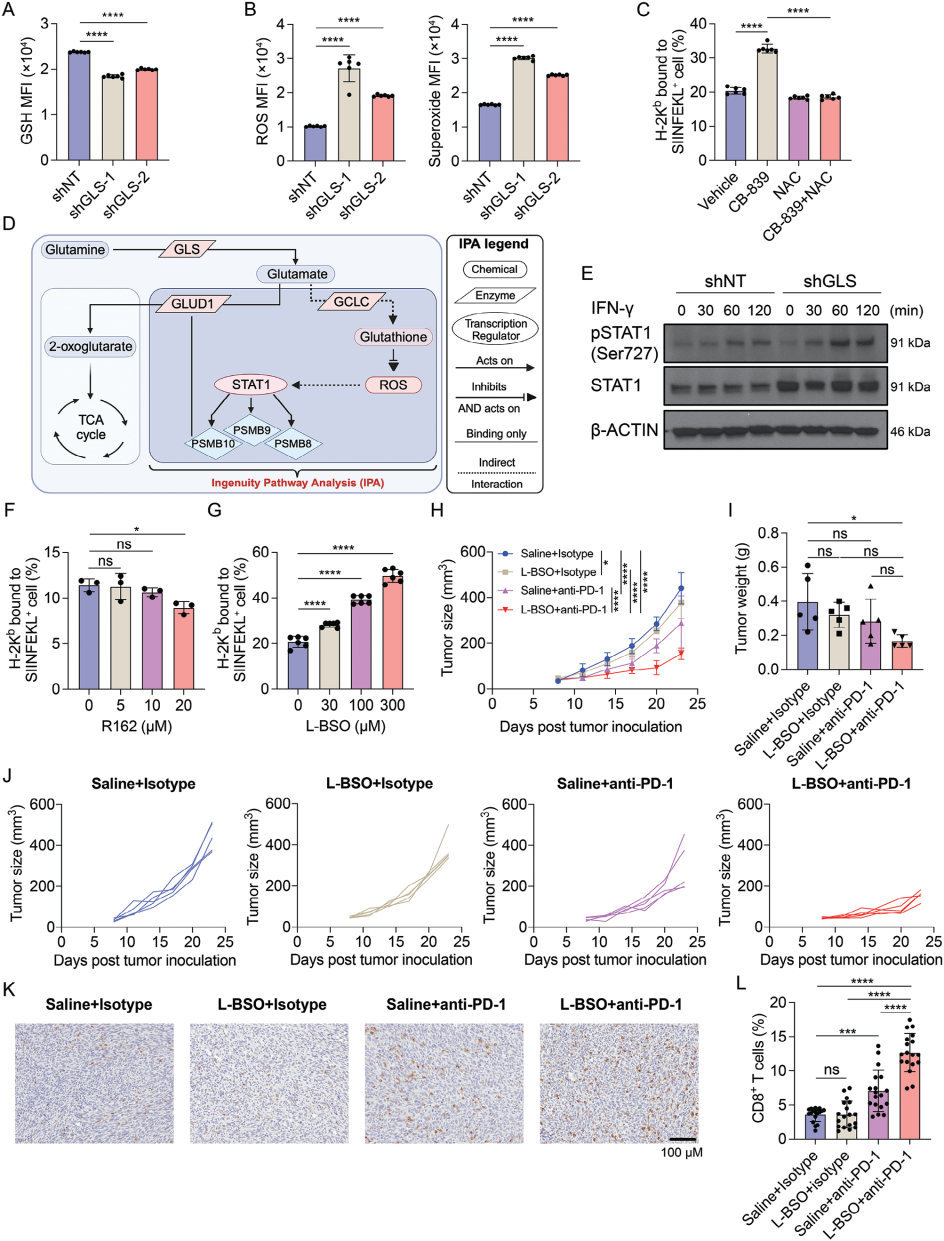
Targeting Glu‐GSH flux enhances tumor antigen presentation and sensitizes CRC to anti‐PD‐1 therapy. A,B) GSH levels (A) and ROS and superoxide levels (B) were determined by flow cytometry in shNT and shGLS‐expressing MC38 cells. Data were analyzed using One‐way ANOVA and Dunnett's multiple comparisons test and presented as mean ± SD (n = 6). C) H‐2K^b^ mediated OVA presentation levels in MC38‐OVA cells treated with CB‐839 and/or NAC were determined by flow cytometry. Data were analyzed using one‐way ANOVA and Tukey's multiple comparisons test and presented as mean ± SD (n = 6). D) Ingenuity Pathway Analysis (IPA) pathway builder was used to construct the signaling pathways that connect ROS to the immunoproteasomes. The illustration was created with BioRender.com. E) Phosphorylated STAT1 and total STAT1 protein levels were determined using Western blotting in shNT and shGLS MC38 cells treated with IFN‐γ at the indicated time points, and β‐ACTIN was used as a loading control. F) H‐2K^b^ mediated OVA presentation levels in MC38‐OVA cells treated with R162 at the indicated doses were determined by flow cytometry. Data were analyzed using one‐way ANOVA and Dunnett's multiple comparisons test and presented as mean ± SD (n = 3), G) H‐2K^b^ mediated OVA presentation levels in MC38‐OVA cells treated with L‐BSO at indicated doses were determined and analyzed as in F). Data were presented as mean ± SD (n = 6). H–J) MC38 tumor growth in C57BL/6J mice. MC38 cells were inoculated subcutaneously into C57BL/6 mice. The tumor‐bearing mice were treated as indicated. The tumor sizes were monitored (H). The tumor weights (I) were taken at the endpoint. The growth curve of each tumor is shown in (J). (H) was analyzed using two‐way ANOVA and Tukey's multiple comparisons test, and (I) was analyzed using one‐way ANOVA and Tukey's multiple comparisons test, data were presented as mean ± SD (n = 5). K,L) Immunohistochemical staining of CD8 and the quantification in the tumors from the indicated four groups. One representative image from each group was shown. The quantification was analyzed using Image J on 18 images of each group. Data were analyzed using one‐way ANOVA, and Tukey's multiple comparisons test, presented as mean ± SD.

The Glu‐GSH flux is important for maintaining the redox balance by fueling the GSH production.^[^ ^10b^ ^]^ Previous studies have found that knockdown of GLS leads to GSH depletion and causes the accumulation of reactive oxygen species (ROS) in tumor cells.^[^ ^38^ ^]^ We hypothesized that glutamine depletion may inhibit Glu‐GSH flux, and thus elevate the level of ROS, leading to increased tumor antigen presentation and anti‐tumor immune response. As expected, the knockdown or pharmacological inhibition of GLS dramatically decreased intracellular GSH levels in both murine and human CRC cell lines (**Figure** **7**A; Figure S7A–D, Supporting Information). Accordingly, both ROS and superoxide were elevated upon GLS inhibition (Figure 7B; Figure S7A–D, Supporting Information). The presented OVA antigen level on the cell surface was also upregulated upon CB‐839 treatment, while this upregulation was completely abolished with the treatment of an antioxidant, N‐acetyl cysteine (NAC) (Figure 7C). Consistent with the above results, the upregulation of immunoproteasome genes induced by GLS knockdown or inhibition was also abolished by NAC treatment (Figure S7E,F, Supporting Information), suggesting a critical role of ROS in connecting Glu‐GSH flux and MHC‐I antigen presentation.

We next asked whether an enforced increase of ROS in tumor cells can induce the MHC‐I antigen presentation. We treated the MC38 cells with hydrogen peroxide (H_2_O_2_), a non‐radical ROS, and determined the cell surface H‐2K^b^ bound to SIINFEKL level. Our results showed that the H_2_O_2_ treatment enhanced the MHC‐I antigen presentation in MC38 cells in a dose‐dependent manner (Figure S7G, Supporting Information), while the NAC treatment fully eliminated the effects (Figure S7H, Supporting Information), suggesting that transient ROS induction increased the MHC‐I antigen presentation of tumor cells.

To dissect the mechanism by which the ROS signaling regulates immunoproteasome gene expression, we employed QIAGEN Ingenuity Pathway Analysis (IPA, QIAGEN Inc., https://digitalinsights.qiagen.com/IPA) to construct the biological pathway.^[^ ^39^ ^]^ As shown in Figure 7D, the IPA fully constructed the pathways highlighting the potential role of STAT1 in connecting ROS with immunoproteasome genes based on published studies. It has been reported that ROS is associated with STAT1 activation via tyrosine phosphorylation.^[^ ^40^ ^]^ Different types of protein kinases may be activated by ROS and mediate the phosphorylation of STAT1.^[^ ^41^ ^]^ This construction was in line with the mRNA‐seq results that the response to IFN‐γ was enhanced upon GLS depletion in the GO pathway analysis (Figure 4A). Notably, numerous studies have reported that the JAK/STAT signaling pathway can regulate immunoproteasome gene expression.^[^ ^35^, ^42^ ^]^ To validate the IPA construction, we treated control and GLS‐KD MC38 cells with mouse IFN‐γ. Based on the level of phosphorylated STAT1 (Ser727), we observed that GLS knockdown remarkably enhanced the JAK/STAT signaling compared to the control group (Figure 7E). Together, these findings indicate that the elevated ROS mediated by Glu‐GSH flux inhibition promotes immunoproteasome gene expression by activating the JAK/STAT pathway in tumor cells.

### 2.7 Targeting Glu‐GSH Flux by GCLC Inhibitor Sensitizes CRC to Immune Checkpoint Blockade Therapy

The metabolic flux analysis showed that the Glu‐GSH flux (M27), but not the Glu‐2‐OG flux (M28), plays an important role in regulating T cell cytotoxicity (Figure 6). To validate it, we sought to determine whether inhibiting GCLC (M27) or GLUD1 (M28) increases MHC‐I antigen presentation in tumor cells. As expected, inhibiting GLUD1 with R162, a potent GLUD1 inhibitor from a group of purpurin derivatives,^[^ ^43^ ^]^ did not increase the OVA antigen presentation level (Figure 7F). However, inhibiting the Glu‐GSH flux by L‐Buthionine‐(S,R)‐sulfoximine (L‐BSO), a cell‐permeable irreversible inhibitor of GCLC,^[^ ^44^ ^]^ greatly enhanced the MHC‐I antigen presentation in a dose‐dependent manner (Figure 7G). The L‐BSO treatment upregulated the expression of immunoproteasome genes in a ROS‐dependent manner as the increase can be fully abolished with NAC treatment (Figure S7I, Supporting Information). To further validate the role of both metabolic fluxes in regulating tumor antigen presentation, we performed the metabolite supplementation assay. Compared to the complete medium, the level of H‐2K^b^ bound to SIINFEKL on the surface of MC38 cells cultured in the glutamine‐free medium was significantly increased, and supplementation with glutamate (an upstream metabolite of GCLC and GLUD1) or NAC abolished this effect. However, supplementation of 2‐OG (a downstream metabolite of GLUD1) did not affect tumor antigen presentation under glutamine deficiency (Figure S7J, Supporting Information). These results strongly demonstrate that the Glu‐GSH flux plays an essential role in regulating antigen presentation in CRC cells. Moreover, the analysis of the TCGA dataset revealed negative correlations between the expression of *GCLC* and the expression of both MHC‐I and MHC‐II antigen presentation genes, whereas the expression of *GLUD1* did not show such correlations (Figure S8, Supporting Information). Therefore, we reasoned that blocking the Glu‐GSH flux using a GCLC inhibitor would increase the antigen presentation in tumor cells and enhance the efficacy of ICB therapy in treating CRC. To test it, we treated the MC38 tumor‐bearing mice with L‐BSO and the anti‐PD‐1 antibody. Both L‐BSO monotherapy and the combinatorial therapy of L‐BSO plus anti‐PD‐1 antibody did not show any organ toxicity in mice (Figure S9, Supporting Information), indicating the tolerance of mice to the treatments. Although L‐BSO monotherapy did not significantly inhibit tumor growth, the combinatorial therapy exhibited significantly greater tumor inhibition compared to the anti‐PD‐1 antibody monotherapy (Figure 7H‐J). Immunohistochemical staining of CD8 on the tumor sections showed that the combinatorial treatment greatly enhanced CD8^+^ T cell tumor infiltration (Figure 7K,L), suggesting an enhanced anti‐tumor immunity effect by combining the Glu‐GSH flux inhibitor and immune checkpoint inhibitor.

## 3 Discussion

Significant advances in cancer immunology have yielded a multitude of novel immunotherapies that enhance the potency of immune responses against tumors. These therapies are mainly designed to either stimulate targeted immune cell responses or counteract immune suppressive signals generated by cancer cells and their microenvironment. In addition to therapeutic antibodies and immune system modulators, adoptive cell transfer, and immune checkpoint inhibitors represent highly promising strategies for cancer treatment.^[^ ^45^ ^]^ However, despite the great achievements in ICB therapies focusing on PD‐1, CTLA‐4, and PD‐L1, many patients with solid tumors fail to experience durable clinical benefits. Currently, ICB therapy is only effective in a minority of CRC patients. The genomic events in CRC tumors appear to dictate the eligibility and response of patients to ICB. However, recent studies showed that MSI‐H or dMMR on its own may not be sufficient to deliver the response to ICB.^[^ ^46^ ^]^ The underlying mechanisms responsible for therapeutic resistance against ICB often involve diminished levels of tumor‐infiltrating lymphocytes, immunosuppressive cell populations, and unfavorable TME.^[^ ^47^ ^]^

Glutamine metabolism is essential to cancer cells, as it fuels TCA cycle intermediates, mediates reduced glutathione formation, and sustains mitochondrial membrane integrity in the proliferating cells.^[^ ^10^, ^13^, ^16^ ^]^ Several small‐molecule inhibitors against GLS have been developed and tested, such as BPTES, CB‐839, and Compound 968. Among these, CB‐839 exhibits exceptional potency and selectivity as a GLS inhibitor. Extensive testing has demonstrated its efficacy in inhibiting growth across various cancer cell types.^[^ ^20^, ^48^ ^]^ Instead of targeting GLS, a glutamine antagonist named 6‐diazo‐5‐oxo‐L‐norleucine (DON) has also been investigated. DON effectively inhibits a wide range of enzymes that use glutamine as a substrate. This blockade of glutamine metabolism by DON resulted in reduced levels of hypoxia, acidosis, and nutrient depletion in the TME, which subsequently conditions effector T cells toward a long‐lasting and highly activated phenotype.^[^ ^49^ ^]^ Glutamine restriction has also been shown to promote memory differentiation and prevent exhaustion of CD8^+^ T cells in vivo.^[^ ^50^ ^]^ Targeting tumor metabolic pathways has been reported to enhance ICB therapeutic efficacy,^[^ ^51^ ^]^ and inhibition of glutamine metabolism exhibited synergistic effects in combination with ICB therapy in different mouse tumor models.^[^ ^52^ ^]^ However, it is unclear which downstream pathway or metabolite(s) from glutamine play essential roles in mediating anti‐tumor immune responses.

In this study, we specifically reconstructed the central metabolism network in a subcellular localization resolution and conducted an in‐silico metabolic flux estimation analysis. To the best of our knowledge, this is the first computational capability to estimate the sample‐/cell‐wise metabolic flux of subcellular localization‐specific reactions. Using the metabolic flux analysis, we demonstrated that the Glu‐GSH flux may play a unique role in regulating antitumor immunity. This finding has been validated experimentally using in vitro and in vivo models included in this study and we further explored the therapeutic potential by targeting this flux. The discovery underscores the power of intervening metabolic pathways in combination with checkpoint blockade immunotherapy.

Furthermore, we found that transiently elevating ROS production by targeting Glu‐GSH flux could activate immunoproteasome activity and increase MHC‐I antigen presentation in CRC cells, driving CD8^+^ T cell‐mediated anti‐tumor immune responses. This finding supports the potential application of tackling ROS in cancer immunotherapy.^[^ ^53^ ^]^ Tumor mutational burden (TMB) and an expression signature of the antigen processing and presenting machinery have been used as effective tumor‐inherent biomarkers to predict cancer immunotherapy response.^[^ ^54^ ^]^ In this study, we have demonstrated that more antigen‐specific CD8^+^ T cells were recruited to the TME upon GLS inhibition, those T cells were also more active and showed less exhaustion compared to the control group (Figure 3). Importantly, reduced GLS activity in tumor cells drove an enriched progenitor exhausted T cell phenotype, which can be reinvigorated by ICB.^[^ ^55^ ^]^ Our data suggest that enhanced antigen presentation by tumor cells can lead to these improved T cell responses. Interestingly, the only patient sample that did not benefit from CB‐839 treatment showed no difference in the immunoproteasome genes (Figure 5J). The reasons underlying the change of the T cells are possibly due to the enhanced antigen presentation in tumor cells when inhibiting glutamine metabolism. This is in line with previous findings showing increased anti‐tumor T cell‐mediated responses upon restoring the tumoral antigen presentation.^[^ ^8^ ^]^ In the meantime, a significant decrease in MDSCs was also found in the GLS‐KD tumors (Figure 3A). This observation is consistent with the reduced T cell exhaustion identified in the same tumors. A similar finding was also reported in a recent study,^[^ ^52b^ ^]^ showing that glutamine antagonism reduces MDSCs by increasing cell death and inhibiting tumor CSF3 secretion. However, we did not see changes in the infiltration of the MDSC population after GLS inhibition in the MC38‐derived orthotopic tumors, reflecting the different immune microenvironments between the two models.

In summary, our study demonstrates that tumor antigen presentation is dramatically affected by the Glu‐GSH flux. Moreover, therapeutic targeting of this flux using pharmacological inhibitors was able to sensitize CRC to ICB therapy. Therefore, this study provides evidence of the mechanisms by which metabolic interventions can alter tumor immunotherapy and potentially allow for more patients to benefit from these treatment regimens.

## 4 Experimental Section

### Chemicals and Reagents

Telaglenastat (CB‐839, Cat#: HY‐12248), L‐Buthionine‐(S,R)‐sulfoximine (L‐BSO, Cat#: HY‐106376A), and R162 (Cat#: HY‐103096) were purchased from MedChemExpress. N‐Acetyl‐L‐cysteine (NAC, Cat#: A7250‐10G) was purchased from Sigma–Aldrich. TRIzol Reagent (Cat#: 15 596 018) was purchased from Invitrogen. Hydrogen peroxide 3% w/v (Cat#: H312‐500) was purchased from Fisher Scientific. Harris Hematoxylin Solution, Modified (Cat#: HHS32‐1L), and citrate buffer (Cat#: C9999‐10 000 mL) were purchased from Sigma–Aldrich. DMEM, high glucose medium (Cat#: 10‐013‐CV) and RPMI 1640 medium (Cat#: 10‐041‐CV), PBS 1X (Cat#: 21‐040‐CV) 0.25% Trypsin EDTA 1X (Cat#: 25‐053‐CI) were purchased from CORNING. McCoy's 5A Medium (Cat#: M9309‐500ML) and DMEM/F‐12 Medium (Cat#: D8437‐500ML) were purchased from Sigma–Aldrich. Fetal Bovine Serum (Cat#: 26140‐079) and Opti‐MEM Reduced Serum Medium (Cat#: 312985‐070) were purchased from Gibco. Penicillin‐Streptomycin Solution (Cat#: SV30010) was purchased from Hyclone.

### Cell Lines

293T, CT26, HCT116, SW480, and MDA‐MB‐231 were obtained from ATCC. MC38 was obtained as a gift from Patrick Hwu at the MD Anderson Cancer Center, Houston, Texas. CT26 cells were cultured in RPMI‐1640 medium, 293T and MC38 cells were cultured in DMEM, HCT116 cells were cultured in McCoy's 5A medium (modified), and MBA‐MD‐231 and SW480 cells were cultured in DMEM/F‐12 medium. All cell culture media were supplemented with 10% fetal bovine serum and 1X penicillin/streptomycin. All cell lines were cultured at 37 °C in a 5% CO_2_ incubator and were routinely tested for mycoplasma contamination.

### Human Samples

Colorectal cancer (CRC) patient samples were obtained from the Tissue Procurement and Distribution Core of Indiana University Simon Comprehensive Cancer Center (IUSCCC) and the experiments complied with the relevant regulatory standard. Details of patient samples are provided in Table S1 (Supporting Information).

### Data Resources

*CRC bulk transcriptomics data*: TCGA RNA‐seq data (FPKM), GSE14333, GSE29621, GSE38832, GSE17536, GSE33113, GSE39572, GSE2019, GSE37892.

*CRC scRNA‐seq data*: GSE81861. Cell‐type annotation was provided by the original work (PMID: 28 319 088).

*Cell line data*: Transcriptomics data of cell lines of immune and CRC cells were previously collected and summarized.^[^ ^18^, ^56^ ^]^

### Deconvolution, Correlation, and Cell Type‐Specific Expression Analysis

Immune cell deconvolution analysis was conducted by using the ICTD algorithm (Inference of Cell Type and Deconvolution). ICTD is a reference deconvolution method. The cytotoxicity level of CD8^+^ T cell was predicted by $\frac{total\;cytotoxic\;level}{total\;CD8^{+}\;T\;cell\;abundance}$, which has been previously defined.^[^ ^18^, ^57^ ^]^ The cytotoxicity level of CD8^+^ T cell was computed for each sample in each of the nine CRC bulk transcriptomics data. Pearson Correlation Coefficients between the cytotoxicity level of CD8^+^ T cell and gene expression level of 3021 human enzyme and transporter genes collected from KEGG^[^ ^58^ ^]^ and the Transporter Classification Database.^[^ ^59^ ^]^ The markers used to define T cells are: “CD3E, CD2, CD3G, CD3D, SIRPG, CD6, TIGIT”, and the markers used to define cytotoxicity are: “CD8A, SLA2, NKG7, PRF1, GZMA, GZMH”. The enzyme and transporter genes were further ranked by the averaged Pearson Correlation Coefficients derived from the nine datasets in increasing order. Cell type‐specific expression of GLS was computed and visualized directly by using the normalized expression level of the gene or probe in the scRNA‐seq and cell line data.

### Reconstruction of Central Metabolism Network in Subcellular Localization

We collected the metabolic reactions related to glucose, glutamate, glutamine, and glutathione metabolism from the KEGG database and manually curated the subcellular localization information of each reaction and enzyme based on the previously curated metabolic network.^[^ ^37^ ^]^ The reconstructed central metabolic network includes 31 reaction modules, 16 intermediate metabolites, 15 end metabolites, and 253 genes in the cytosol, mitochondria, and extracellular regions. The reconstructed network includes six major pathways, namely glycolysis, upper and lower parts of TCA cycle, glutaminolysis, glutamine and glutamate metabolism, and glutathione metabolism,^[^ ^60^ ^]^ and three minor branches, namely Glyceraldehyde 3‐phosphate (G3P) to nucleotide synthesis, and 3‐Phospho‐D‐glycerate (3PD) to serine synthesis, and aspartate‐malate shuttle. The curated network was represented in a directed factor graph as described in ref. [18]. Detailed lists of reactions and genes in this network are given in Tables S2 and S3 (Supporting Information).

### Metabolic Flux Analysis

While the details of the scFEA method are given in,^[^ ^37^ ^]^ we outline the key ideas of the algorithm. scFEA is based on a novel graph neural network architecture to model the sample‐wise metabolic flux of each module by using their transcriptomic profiles. The inputs to scFEA include (1) gene expression data and (2) a factor graph‐based representation of the metabolic map. Specifically, we formulate a metabolic as a directed factor graph, where each module represents a factor, and each intermediate compound is a variable node carrying a likelihood function describing its flux balance.^[^ ^37^ ^]^

Denote *FG*(*C*, *R*,  *E*  =  {*E*_*C* → *R*_, *E*_*R* → *C*_}) as the factor graph, where *C* is the set of metabolites, *R* is the set of metabolic modules, *E*_*C* → *R*_ and *E*_*R* → *C*_ represent direct edges from module to metabolite and from metabolite to module, respectively. For each intermediate metabolite *C_k_* in the network, define the set of modules consuming and producing each *C_k_* as $F_{in}^{C_{k}}=\{R_{m}|\;\left(R_{m}\to C_{k}\right)\in E_{C\to R}\}$ and $F_{out}^{C_{k}}=\{R_{m}|\;\left(C_{k}\to R_{m}\right)\in E_{R\to C}\}$. For transcriptomics data containing *N* samples, denote $G^{m}=\left\{G_{1}^{m},\text{…},G_{i_{m}}^{m}\right\}$ as the genes involved in the module *R_m_*, $G_{j}^{m}=\left\{G_{1,j}^{m},\text{…},G_{i_{m},j}^{m}\right\}\;$ as their expression and *Flux*_*m*,*j*_ as the flux of the module *m* in the cell or sample *j*. We model $Flu\;x_{m,j}=f_{nn}^{m}\;\left(G_{j}^{m}|\;\theta_{m}\right)$ as a multi‐layer fully connected neural network with the input $G_{j}^{m}$, where ***θ***_***m***_ denotes the parameters of the neural network. Then the ***θ***_***m***_ and cell‐ or sample‐wise flux *Flux*_*m*,*j*_ are solved by minimizing the following loss function:

(1) $$\begin{array}{ccc} & & L_{0}=\sum_{j=1}^{N}\sum_{k=1}^{K}{\left(\sum_{m\in F_{in}^{C_{k}}}Flux_{m,j}-\sum_{m^{\prime }\in F_{out}^{C_{k}}}Flux_{m^{\prime },j}\right)}^{2}\\ & & \;+\alpha \sum_{j=1}^{N}\sum_{m=1}^{M}\left(\left|Flux_{m,j}\right|-Flux_{m,j}\right)+\beta \sum_{j=1}^{N}{\left(\sum_{m=1}^{M}Flux_{m,j}-TA_{j}\right)}^{2} \end{array}$$

where α and β are hyperparameters, and *TA_j_* is a surrogate for total metabolic activity level of cell or sample *j*, which is assigned as the total expression of metabolic genes in cell or sample *j*. Hence, the first, second, and third terms of *L* correspond to constraints on flux balance, non‐negative flux, and the relative scale of flux, respectively. The above flux estimation model has been validated on two sets of matched scRNA‐seq and bulk cell metabolomics data, simulated scRNA‐seq and fluxome data, and 5 sets of high‐quality tissue transcriptomics or scRNA‐seq datasets.^[^ ^37^ ^]^ The previous robustness analyses suggested that an empirical setting of α  =  1, β  =  0.1, γ  =  1 can guarantee good prediction accuracies of reasonable biological interpretability, with a fast convergence rate.^[^ ^61^ ^]^

### Generation of Ovalbumin Antigen‐Expressing Cells

Lentiviruses carrying a lentiviral full‐length ovalbumin (OVA) ORF expression construct were packaged using psPAX2 (Addgene, Cat#: 12 260) and pMD2.G (Addgene, Cat#: 12 259), and transfection was performed using the linear polyethylenimine, MW25000 (Polysciences, Cat#: 23966‐1). Three days after the lentivirus infection, the MC38 cells were selected with puromycin, and intracellular OVA expression was further confirmed using Western blotting. Single colonies were selected using serial dilution and the MHC‐I‐mediated SIIFEKLE presentation was determined using flow cytometry.

### T Cell Cytotoxicity Assay

The luciferase overexpressed MC38 cells (MC38‐luc) were pre‐loaded with Ovalbumin (257–264) peptide (Sigma–Aldrich, Cat#: S7951‐1MG) at the concentration of 5 µg ml^−1^ for 1 h at 37 °C in DMEM, and then washed with fresh DMEM for three times before seeding into the 96‐well plate. CD8^+^ T cells were isolated from the spleen of OT‐I mice using mouse CD8a Microbeads (Miltenyi Biotec, Cat#: 130‐117‐044) and pre‐treated with Dynabead Mouse T‐Activator CD3/CD28 (Gibco, Cat#: 11452D) in the presence of mouse IL‐2 (BioLegend, Cat#: 575 404) for two days. The OVA peptide‐loaded MC38 cells and CD8^+^ T cells were counted and co‐cultured in a 96‐well plate at the ratios of 1:1 or 1:5 (T cell: tumor cell) for 16 h. A no T cell group was used as a control. After 16 h of co‐culture, the tumor cell killing was determined using the Luciferase Assay System (Promega, Cat#: E1500).

### Human Sample‐Derived Organoids Killing Assay

CRC patient sample‐derived organoids (PDOs) were generated as previously described.^[^ ^62^ ^]^ The CRC tissue autologous CD8^+^ T cells were isolated using human CD8 Microbeads (Miltenyi Biotec, Cat#: 130‐045‐201) and were then pre‐activated with Dynabead Human T‐Activator CD3/CD28 (Gibco, Cat#: 11131D) in the presence of human IL‐2 (PeproTech, Cat#: 200–02) for 2 days before the co‐culture. The organoids between 70 and 150 µm were selected using cell strainers for the autologous CD8^+^ T cells killing assay. The organoids and activated T cells were then co‐cultured in a 24‐well clear flat bottom ultra‐low attachment multiple‐well plate (Costar) at the ratio of 200: 1 (T cell: organoid) for 24 h. The images of the organoids were taken, and the sizes of the organoids were analyzed using Image J software.

### Western Blotting

Total proteins were prepared using the Pierce RIPA Lysis and Extraction Buffer (Thermo Scientific, Cat#: 89 000) with the PhosSTOP EASYpack (Roche, Cat#: 0 490 684 5001) and cOmplete Protease Inhibitor Cocktail (Roche, Cat#: 11 697 498 001) added to inhibit phosphatases and proteases. The protein lysates were quantified using the Pierce BCA Protein Assay Kits (Thermo Scientific, Cat#: A55864). Equal amounts of protein (20–40 µg) from each sample in premixed Laemmli Protein Sample Buffer (Bio‐rad, Cat#: 1 610 747) were loaded to Bio‐Rad Mini‐PROTEAN TGX Precast Gels for electrophoresis and then transferred onto PVDF membranes for immunoblotting. The antibodies used in Western Blotting are shown in Table S4 (Supporting Information).

### Flow Cytometry

The flow cytometry was performed on the LSR Fortessa X‐20 or LSR Fortessa (BD Biosciences) systems, and the data were analyzed using FlowJo version 10. The SYTOX Blue (Invitrogen) or eBioscience Fixable Viability Dye eFluor 506 (Invitrogen) were used to stain live/dead cells. 10% normal goat serum, 10% FBS, and CD16/32 antibody were used to block non‐specific signals in the assay. The cells were fixed and permeabilized using the BD Cytofix/Cytoperm fixation permeabilization kit for the staining of intracellular markers. The antibodies used in flow cytometry are shown in Table S4 (Supporting Information) and were used in several combinations. A mouse tumor dissociation kit (Miltenyi Biotec, Cat#: 130‐096‐730) was used to dissociate mouse tumors for flow cytometry acquisition.

### Quantitative RT‐PCR

Total RNA was isolated using a Direct‐zol RNA Miniprep kit (ZYMO RESEARCH, Cat#: R2052), and cDNA was synthesized using qScript cDNA SuperMix Kit (Quantabio, Cat#: 95048–500). The quantitative PCR was performed using SYBR Green PCR Master Mix (Applied Biosystems, Cat#: 4 309 155) with gene‐specific primers according to the manufacturer's instructions. The primer sequences are shown in Table S5 (Supporting Information).

### Immunohistochemistry

Tissue slides were deparaffinized and rehydrated. The antigen retrieval was performed using citrate buffer. To block endogenous peroxidase activity, the slides were incubated with 3% hydrogen peroxide for 10 min. After 1 h of blocking with 5% normal goat serum, the slides were incubated with anti‐CD8α antibody or anti‐granzyme B antibody (see Table S4, Supporting Information) at 4 °C overnight. After washing, the slides were incubated with 4Plus biotinylated anti‐rabbit IgG and then incubated with 4Plus streptavidin HRP label (BIOCARE, Cat#: HP604H). The slides were developed using the DAB peroxidase substrate kit (Vector Laboratories, Cat#: SK4100) according to the manufacturer's instructions. The counterstaining was carried out using Harris Modified Hematoxylin.

### Cell Proliferation Assay

An equal number of cells (control and GLS‐KD) were seeded in 96‐well plates and cultured for 4 to 5 days. The cells were fixed with 10% buffered formalin phosphate and then stained with 0.1% crystal violet (dissolved in 10% methanol). After staining, washing, and drying, add 150 µl of methanol to each well and incubate with a lid for 20 min at room temperature, and the absorbance of each well was measured at 570 nm with a plate reader.

### shRNA Interference

Human and mouse glutaminase MISSION® shRNA Bacterial Glycerol Stocks were purchased from Sigma–Aldrich. The shRNAs used in the current study were validated by Sigma–Aldrich to have a knockdown efficiency of more than 80%. Lentiviruses were packaged using psPAX2 and pMD2.G and transfection was performed using the linear polyethylenimine. The clone IDs and shRNA sequences are Human *GLS* shRNA‐1: TRCN0000051134, 5′‐ CCATAAGAATCTTGATGGATT‐3′; Human *GLS* shRNA‐2: TRCN0000051135, 5′‐GCACAGACATGGTTGGTATAT‐3′; Mouse *Gls* shNRA‐1: TRCN0000253163, 5′‐AGAAAGTGGAGATCGAAATTT‐3′; Mouse *Gls* shRNA‐2: TRCN0000253167, 5′‐GAGGGAAGGTTGCTGATTATA‐3′. The pLKO.1 puro (Addgene, Cat#: 8453) was used as a control.

### Bulk mRNA Sequencing and Analysis

MC38 cells were seeded in 6‐well plates and treated with vehicle (DMSO) or 2 µM CB‐839 for 2 days. Then all the cells were collected and lysed with TriZol reagent. The total RNA was extracted using the ZYMO Direct‐zol RNA miniprep kit according to the manufacturer's instructions. All the RNA samples were then quality‐checked before the sequencing. The reads were mapped to the mouse genome mm10 using STAR (v2.7.2a). RNA‐seq aligner with the following parameter: ′'–outSAMmapqUnique 60″. Uniquely mapped sequencing reads were assigned to the GENCODE M22 gene using featureCounts (v1.6.5) with the following parameters: “‐s 2 –p –Q 10 ‐O”. The data was filtered using read count >10 in at least 3 of the samples, normalized using the TMM (trimmed mean of M values) method, and subjected to differential expression analysis using edgeR (v3.20.8). Gene ontology and KEGG pathway functional analysis were performed on differential expression genes with a false discovery rate cut‐off of 0.05 and the absolute value of log2 of fold change cut‐off of 0.5 using DAVID.

### Tumor Implantation and Treatment

Eight‐week‐old female and/or male NU/J mice, C57BL/6J, and BALB/c mice were used in the in vivo tumor studies. For tumor growth experiments, 2 × 10^5^ MC38 cells (control and GLS‐KD) or 1 × 10^5^ CT26 cells (control and GLS‐KD) in PBS were subcutaneously injected into the flank. Tumor size was measured every three days using a caliper after the tumors reached ≈50 mm^3^. For the orthotopic survival experiment, 1 × 10^5^ MC38 cells (control and GLS‐KD) were injected in a volume of 50 µl PBS using a 30‐gauge needle into the cecal wall. In the combinatorial treatment experiment, 2 × 10^5^ MC38 cells in PBS were subcutaneously injected into the flank of the C57BL/J mice. 10 days after injection, the mice were randomly divided into four groups followed by vehicle (isotype control and saline), L‐BSO (200 mg k^−1^g, i.p., once a day), and anti‐PD‐1 antibody (200 ug per mouse, i.p., once every three days) treatment. Tumor size was measured every three days using a caliper, and tumor volume was calculated using the standard formula: 0.5 × L × W^2^, where L is the longest diameter and W is the shortest diameter. Sample sizes were chosen to ensure they were sufficient for statistical comparison between different groups and were provided in the corresponding figure legend. At the experimental endpoint, tumors or organs were harvested for the following assays. All the mice used in this study were housed under pathogen‐free conditions, with the ambient room temperature 22 ± 2 °C, 12/12 h light/dark cycle (lights on at 7:00 a.m.), and 30–70% relative humidity in the Laboratory Animal Resource Center (LARC) at Indiana University School of Medicine. Any mouse with a tumor size ≥1500 mm^3^ needs to be euthanized according to the approved animal protocol.

For the in vivo CD8^+^ T cell depletion experiment, control and GLS‐KD MC38 cells (2 × 10^5^ MC38 cells per mouse) were injected subcutaneously into the flanks of the C57BL/J mice. To deplete the CD8^+^ T cells in vivo, the anti‐CD8 (clone 53–6.7; Bio X Cell) and the rat IgG2a isotype control (clone 2A3, Bio X Cell) were administered 2 days before tumor cell inoculation and continued until the end of the experiment. Antibodies were administrated at a dose of 100 µg per mouse, 3 times a week.

### Immunoproteasome Activity Assay

The immunoproteasome activity was determined using the Immunoproteasome Activity Fluorometric Assay Kit I (UBPBio, Cat#: J4160) according to the manufacturer's instructions. Briefly, the cells were collected, re‐suspended in ice‐cold cell lysis buffer, and were briefly sonicated. The supernatants were collected after centrifugation, and protein concentrations were determined using a BCA assay kit. Then the supernatants were diluted with assay buffer and mixed with Ac‐ANW‐AMC substrate. The AMC fluorescence was determined using a plate reader immediately after adding the substrate. An MG132‐treated sample was used as a negative control in the assay.

### GSH and ROS Determination

The cellular GSH levels were measured using the Intracellular glutathione (GSH) Detection Assay Kit (Abcam, Cat#: ab112132), ROS levels were measured using the Cellular ROS/Superoxide Detection Assay Kit (Abcam, Cat#: ab139476) according to the manufacturer's instructions. Briefly, the cells were collected and incubated with Thiol green dye (GSH detection), or ROS/Superoxide Mix for 30 min. The cells were then washed and analyzed using a flow cytometer.

### Quantitation and Statistical Analysis

Statistical analyses were performed using GraphPad Prism 9. For normally distributed data and comparisons between two groups, the significance was calculated using unpaired two‐tailed Student's t‐tests. Comparisons between three or more groups were performed using the One‐way ANOVA or Two‐way ANOVA. Log‐rank test was used to statistically compare the mouse survival curves of different groups. Throughout the paper, significance was determined at the following cutoff points: not significant (ns), *p* >0.05; *p < 0.05; **p < 0.01; ***p < 0.001; ****p < 0.0001.

### Study Approval

All the animal experiments have been approved by the Institutional Animal Care and Use Committee of Indiana University School of Medicine.

### Patient Consent

The informed consent was obtained from patients before tissue collection.

## Conflict of Interest

The authors declare no conflict of interest.

## Author Contributions

T.Y. and K.V.d.J. contributed equally to this work. T.Y., K.V.d.J., C.Z., and X.Z. conceived and designed the study. T.Y., K.V.d.J., H.Z., Z.Z., S.S., K.M.S., and H.E. performed experiments and data analysis. Y.L., M.O., S.C., J.W., C.Z., and X.Z. provided critical reagents and/or insights. S.L. and J.W. analyzed the mRNA‐seq data. G.E.S. assessed the H&E staining of the organ tissues. T.Y., K.V.d.J., C.Z., and X.Z. wrote the original draft of the manuscript. T.Y., K.V.d.J., M.O., C.Z., and X.Z. revised the manuscript. C.Z. and X.Z. supervised the study.

## Supporting information



Supporting Information

## Data Availability

The data that support the findings of this study are available from the corresponding author upon reasonable request.

## References

[1] R. L. Siegel , N. S. Wagle , A. Cercek , R. A. Smith , A. Jemal , CA Cancer J Clin. 2023, 73, 233. 36856579 10.3322/caac.21772

[2] a) J. Aparicio , F. Esposito , S. Serrano , E. Falco , P. Escudero , A. Ruiz‐Casado , H. Manzano , A. Fernandez‐Montes , J Clin Med. 2020, 9, 3889; 33265959 10.3390/jcm9123889 PMC7761096

[3] S. Venderbosch , I. D. Nagtegaal , T. S. Maughan , C. G. Smith , J. P. Cheadle , D. Fisher , R. Kaplan , P. Quirke , M. T. Seymour , S. D. Richman , G. A. Meijer , B. Ylstra , D. A. Heideman , A. F. de Haan , C. J. Punt , M. Koopman , Clin. Cancer Res. 2014, 20, 5322. 25139339 10.1158/1078-0432.CCR-14-0332 PMC4201568

[4] L. Marcus , S. J. Lemery , P. Keegan , R. Pazdur , Clin. Cancer Res. 2019, 25, 3753. 30787022 10.1158/1078-0432.CCR-18-4070

[5] S. Jhunjhunwala , C. Hammer , L. Delamarre , Nat. Rev. Cancer 2021, 21, 298. 33750922 10.1038/s41568-021-00339-z

[6] a) K. Yang , A. Halima , T. A. Chan , Nat. Rev. Clin. Oncol. 2023, 20, 604; 37328642 10.1038/s41571-023-00789-4

[7] a) S. Gettinger , J. Choi , K. Hastings , A. Truini , I. Datar , R. Sowell , A. Wurtz , W. Dong , G. Cai , M. A. Melnick , V. Y. Du , J. Schlessinger , S. B. Goldberg , A. Chiang , M. F. Sanmamed , I. Melero , J. Agorreta , L. M. Montuenga , R. Lifton , S. Ferrone , P. Kavathas , D. L. Rimm , S. M. Kaech , K. Schalper , R. S. Herbst , K. Politi , Cancer Discov. 2017, 7, 1420; 29025772 10.1158/2159-8290.CD-17-0593 PMC5718941

[8] a) P. M. K. Westcott , N. J. Sacks , J. M. Schenkel , Z. A. Ely , O. Smith , H. Hauck , A. M. Jaeger , D. Zhang , C. M. Backlund , M. C. Beytagh , J. J. Patten , R. Elbashir , G. Eng , D. J. Irvine , O. H. Yilmaz , T. Jacks , Nat. Cancer 2021, 2, 1071; 34738089 10.1038/s43018-021-00247-z PMC8562866

[9] D. Hanahan , R. A. Weinberg , Cell 2011, 144, 646. 21376230 10.1016/j.cell.2011.02.013

[10] a) T. Yu , T. Dong , H. Eyvani , Y. Fang , X. Wang , X. Zhang , X. Lu , Arch. Biochem. Biophys. 2021, 697, 108659; 33144083 10.1016/j.abb.2020.108659 PMC8638212

[11] a) S. A. Best , P. M. Gubser , S. Sethumadhavan , A. Kersbergen , Y. L. Negron Abril , J. Goldford , K. Sellers , W. Abeysekera , A. L. Garnham , J. A. McDonald , C. E. Weeden , D. Anderson , D. Pirman , T. P. Roddy , D. J. Creek , A. Kallies , G. Kingsbury , K. D. Sutherland , Cell Metab. 2022, 34, 874; 35504291 10.1016/j.cmet.2022.04.003

[12] H. G. Windmueller , A. E. Spaeth , J. Biol. Chem. 1974, 249, 5070; 4605420

[13] L. Yang , S. Venneti , D. Nagrath , Annu. Rev. Biomed. Eng. 2017, 19, 163. 28301735 10.1146/annurev-bioeng-071516-044546

[14] a) R. Shah , S. Chen , Cancers 2020, 12, 2624; 32937954 10.3390/cancers12092624 PMC7565600

[15] a) J. Son , C. A. Lyssiotis , H. Ying , X. Wang , S. Hua , M. Ligorio , R. M. Perera , C. R. Ferrone , E. Mullarky , N. Shyh‐Chang , Y. Kang , J. B. Fleming , N. Bardeesy , J. M. Asara , M. C. Haigis , R. A. DePinho , L. C. Cantley , A. C. Kimmelman , Nature 2013, 496, 101; 23535601 10.1038/nature12040 PMC3656466

[16] a) L. Jin , G. N. Alesi , S. Kang , Oncogene 2016, 35, 3619; 26592449 10.1038/onc.2015.447 PMC5225500

[17] M. D. Buck , R. T. Sowell , S. M. Kaech , E. L. Pearce , Cell 2017, 169, 570. 28475890 10.1016/j.cell.2017.04.004 PMC5648021

[18] W. Chang , C. Wan , X. Lu , S.‐w. Tu , Y. Sun , X. Zhang , Y. Zang , A. Zhang , K. Huang , Y. Liu , X. Lu , S. Cao , C. Zhang , bioRxiv 2019, 10.1101/426593.

[19] Y. Hao , Y. Samuels , Q. Li , D. Krokowski , B. J. Guan , C. Wang , Z. Jin , B. Dong , B. Cao , X. Feng , M. Xiang , C. Xu , S. Fink , N. J. Meropol , Y. Xu , R. A. Conlon , S. Markowitz , K. W. Kinzler , V. E. Velculescu , H. Brunengraber , J. E. Willis , T. LaFramboise , M. Hatzoglou , G. F. Zhang , B. Vogelstein , Z. Wang , Nat. Commun. 2016, 7, 11971. 27321283 10.1038/ncomms11971 PMC4915131

[20] M. I. Gross , S. D. Demo , J. B. Dennison , L. Chen , T. Chernov‐Rogan , B. Goyal , J. R. Janes , G. J. Laidig , E. R. Lewis , J. Li , A. L. Mackinnon , F. Parlati , M. L. Rodriguez , P. J. Shwonek , E. B. Sjogren , T. F. Stanton , T. Wang , J. Yang , F. Zhao , M. K. Bennett , Mol. Cancer Ther. 2014, 13, 890. 24523301 10.1158/1535-7163.MCT-13-0870

[21] P. Korangath , W. W. Teo , H. Sadik , L. Han , N. Mori , C. M. Huijts , F. Wildes , S. Bharti , Z. Zhang , C. A. Santa‐Maria , H. Tsai , C. V. Dang , V. Stearns , Z. M. Bhujwalla , S. Sukumar , Clin. Cancer Res. 2015, 21, 3263. 25813021 10.1158/1078-0432.CCR-14-1200 PMC4696069

[22] K. Van der Jeught , Y. Sun , Y. Fang , Z. Zhou , H. Jiang , T. Yu , J. Yang , M. M. Kamocka , K. M. So , Y. Li , H. Eyvani , G. E. Sandusky , M. Frieden , H. Braun , R. Beyaert , X. He , X. Zhang , C. Zhang , S. Paczesny , X. Lu , JCI Insight. 2020, 5, e136073. 32376804 10.1172/jci.insight.136073 PMC7253019

[23] S. R. Clarke , M. Barnden , C. Kurts , F. R. Carbone , J. F. Miller , W. R. Heath , Immunol. Cell Biol. 2000, 78, 110. 10762410 10.1046/j.1440-1711.2000.00889.x

[24] J. C. Yang , D. Perry‐Lalley , J. Immunother. 2000, 23, 177. 10746543 10.1097/00002371-200003000-00001

[25] Y. Jiang , Y. Li , B. Zhu , Cell Death Dis. 2015, 6, e1792. 26086965 10.1038/cddis.2015.162 PMC4669840

[26] C. U. Blank , W. N. Haining , W. Held , P. G. Hogan , A. Kallies , E. Lugli , R. C. Lynn , M. Philip , A. Rao , N. P. Restifo , A. Schietinger , T. N. Schumacher , P. L. Schwartzberg , A. H. Sharpe , D. E. Speiser , E. J. Wherry , B. A. Youngblood , D. Zehn , Nat. Rev. Immunol. 2019, 19, 665. 31570879 10.1038/s41577-019-0221-9 PMC7286441

[27] B. C. Miller , D. R. Sen , R. Al Abosy , K. Bi , Y. V. Virkud , M. W. LaFleur , K. B. Yates , A. Lako , K. Felt , G. S. Naik , M. Manos , E. Gjini , J. R. Kuchroo , J. J. Ishizuka , J. L. Collier , G. K. Griffin , S. Maleri , D. E. Comstock , S. A. Weiss , F. D. Brown , A. Panda , M. D. Zimmer , R. T. Manguso , F. S. Hodi , S. J. Rodig , A. H. Sharpe , W. N. Haining , Nat. Immunol. 2019, 20, 326. 30778252 10.1038/s41590-019-0312-6 PMC6673650

[28] M. Abd Hamid , H. Colin‐York , N. Khalid‐Alham , M. Browne , L. Cerundolo , J.‐L. Chen , X. Yao , S. Rosendo‐Machado , C. Waugh , D. Maldonado‐Perez , E. Bowes , C. Verrill , V. Cerundolo , C. P. Conlon , M. Fritzsche , Y. Peng , T. Dong , Cancer Immunol. Res. 2020, 8, 203. 31771983 10.1158/2326-6066.CIR-19-0554 PMC7611226

[29] a) O. Khan , J. R. Giles , S. McDonald , S. Manne , S. F. Ngiow , K. P. Patel , M. T. Werner , A. C. Huang , K. A. Alexander , J. E. Wu , J. Attanasio , P. Yan , S. M. George , B. Bengsch , R. P. Staupe , G. Donahue , W. Xu , R. K. Amaravadi , X. Xu , G. C. Karakousis , T. C. Mitchell , L. M. Schuchter , J. Kaye , S. L. Berger , E. J. Wherry , Nature 2019, 571, 211; 31207603 10.1038/s41586-019-1325-x PMC6713202

[30] F. Zhou , Int. Rev. Immunol. 2009, 28, 239. 19811323 10.1080/08830180902978120

[31] A. Subramanian , P. Tamayo , V. K. Mootha , S. Mukherjee , B. L. Ebert , M. A. Gillette , A. Paulovich , S. L. Pomeroy , T. R. Golub , E. S. Lander , J. P. Mesirov , Proc Natl. Acad. Sci. U S A 2005, 102, 15545. 16199517 10.1073/pnas.0506580102 PMC1239896

[32] A. Liberzon , C. Birger , H. Thorvaldsdottir , M. Ghandi , J. P. Mesirov , P. Tamayo , Cell Syst. 2015, 1, 417. 26771021 10.1016/j.cels.2015.12.004 PMC4707969

[33] S. Murata , Y. Takahama , M. Kasahara , K. Tanaka , Nat. Immunol. 2018, 19, 923. 30104634 10.1038/s41590-018-0186-z

[34] M. Basler , C. J. Kirk , M. Groettrup , Curr. Opin. Immunol. 2013, 25, 74. 23219269 10.1016/j.coi.2012.11.004

[35] S. C. Tripathi , H. L. Peters , A. Taguchi , H. Katayama , H. Wang , A. Momin , M. K. Jolly , M. Celiktas , J. Rodriguez‐Canales , H. Liu , C. Behrens , Wistuba II , E. Ben‐Jacob , H. Levine , J. J. Molldrem , S. M. Hanash , E. J. Ostrin , Proc Natl Acad Sci U S A 2016, 113, E1555. 26929325 10.1073/pnas.1521812113 PMC4801290

[36] A. Rouette , A. Trofimov , D. Haberl , G. Boucher , V. P. Lavallee , G. D'Angelo , J. Hebert , G. Sauvageau , S. Lemieux , C. Perreault , Sci. Rep. 2016, 6, 34019. 27659694 10.1038/srep34019 PMC5034284

[37] N. Alghamdi , W. Chang , P. Dang , X. Lu , C. Wan , S. Gampala , Z. Huang , J. Wang , Q. Ma , Y. Zang , M. Fishel , S. Cao , C. Zhang , Genome Res. 2021, 31, 1867. 34301623 10.1101/gr.271205.120 PMC8494226

[38] a) Y. Xiang , Z. E. Stine , J. Xia , Y. Lu , R. S. O'Connor , B. J. Altman , A. L. Hsieh , A. M. Gouw , A. G. Thomas , P. Gao , L. Sun , L. Song , B. Yan , B. S. Slusher , J. Zhuo , L. L. Ooi , C. G. Lee , A. Mancuso , A. S. McCallion , A. Le , M. C. Milone , S. Rayport , D. W. Felsher , C. V. Dang , J. Clin. Invest. 2015, 125, 2293; 25915584 10.1172/JCI75836 PMC4497742

[39] A. Kramer , J. Green , J. Pollard Jr. , S. Tugendreich , Bioinformatics 2014, 30, 523. 24336805 10.1093/bioinformatics/btt703 PMC3928520

[40] A. R. Simon , U. Rai , B. L. Fanburg , B. H. Cochran , Am. J. Physiol. 1998, 275, C1640. 9843726 10.1152/ajpcell.1998.275.6.C1640

[41] a) H. S. Kim , M. S. Lee , Mol. Cell. Biol. 2005, 25, 6821; 16024814 10.1128/MCB.25.15.6821-6833.2005 PMC1190352

[42] a) T. A. Griffin , D. Nandi , M. Cruz , H. J. Fehling , L. V. Kaer , J. J. Monaco , R. A. Colbert , J. Exp. Med. 1998, 187, 97; 9419215 10.1084/jem.187.1.97 PMC2199179

[43] L. Jin , D. Li , G. N. Alesi , J. Fan , H. B. Kang , Z. Lu , T. J. Boggon , P. Jin , H. Yi , E. R. Wright , D. Duong , N. T. Seyfried , R. Egnatchik , R. J. DeBerardinis , K. R. Magliocca , C. He , M. L. Arellano , H. J. Khoury , D. M. Shin , F. R. Khuri , S. Kang , Cancer Cell 2015, 27, 257. 25670081 10.1016/j.ccell.2014.12.006 PMC4325424

[44] a) J. P. Fruehauf , S. Zonis , M. al‐Bassam , A. Kyshtoobayeva , C. Dasgupta , T. Milovanovic , R. J. Parker , A. C. Buzaid , Pigment Cell Res. 1997, 10, 236; 9263331 10.1111/j.1600-0749.1997.tb00490.x

[45] a) A. Ribas , J. D. Wolchok , Science 2018, 359, 1350; 29567705 10.1126/science.aar4060 PMC7391259

[46] a) C. Mowat , S. R. Mosley , A. Namdar , D. Schiller , K. Baker , J. Exp. Med. 2021, 218, e20210108; 34297038 10.1084/jem.20210108 PMC8313406

[47] a) S. Spranger , T. F. Gajewski , Nat. Rev. Cancer 2018, 18, 139; 29326431 10.1038/nrc.2017.117 PMC6685071

[48] N. M. Zacharias , N. Baran , S. S. Shanmugavelandy , J. Lee , J. V. Lujan , P. Dutta , S. W. Millward , T. Cai , C. G. Wood , D. Piwnica‐Worms , M. Konopleva , P. K. Bhattacharya , Mol. Cancer Ther. 2019, 18, 1937. 31387889 10.1158/1535-7163.MCT-18-0985 PMC7080291

[49] R. D. Leone , L. Zhao , J. M. Englert , I. M. Sun , M. H. Oh , I. H. Sun , M. L. Arwood , I. A. Bettencourt , C. H. Patel , J. Wen , A. Tam , R. L. Blosser , E. Prchalova , J. Alt , R. Rais , B. S. Slusher , J. D. Powell , Science 2019, 366, 1013. 31699883 10.1126/science.aav2588 PMC7023461

[50] S. Nabe , T. Yamada , J. Suzuki , K. Toriyama , T. Yasuoka , M. Kuwahara , A. Shiraishi , K. Takenaka , M. Yasukawa , M. Yamashita , Cancer Sci. 2018, 109, 3737. 30302856 10.1111/cas.13827 PMC6272119

[51] a) J. E. Bader , K. Voss , J. C. Rathmell , Mol. Cell 2020, 78, 1019; 32559423 10.1016/j.molcel.2020.05.034 PMC7339967

[52] a) D. N. Edwards , V. M. Ngwa , A. L. Raybuck , S. Wang , Y. Hwang , L. C. Kim , S. H. Cho , Y. Paik , Q. Wang , S. Zhang , H. C. Manning , J. C. Rathmell , R. S. Cook , M. R. Boothby , J. Chen , J. Clin. Invest. 2021, 131, e140100; 33320840 10.1172/JCI140100 PMC7880417

[53] B. Perillo , M. Di Donato , A. Pezone , E. Di Zazzo , P. Giovannelli , G. Galasso , G. Castoria , A. Migliaccio , Exp. Mol. Med. 2020, 52, 192. 32060354 10.1038/s12276-020-0384-2 PMC7062874

[54] S. Wang , Z. He , X. Wang , H. Li , X. S. Liu , Elife 2019, 8, 49020. 10.7554/eLife.49020 PMC6879305 31767055

[55] a) A. O. Kamphorst , A. Wieland , T. Nasti , S. Yang , R. Zhang , D. L. Barber , B. T. Konieczny , C. Z. Daugherty , L. Koenig , K. Yu , G. L. Sica , A. H. Sharpe , G. J. Freeman , B. R. Blazar , L. A. Turka , T. K. Owonikoko , R. N. Pillai , S. S. Ramalingam , K. Araki , R. Ahmed , Science 2017, 355, 1423; 28280249 10.1126/science.aaf0683 PMC5595217

[56] X. Lu , S. W. Tu , W. Chang , C. Wan , J. Wang , Y. Zang , B. Ramdas , R. Kapur , X. Lu , S. Cao , C. Zhang , Brief Bioinform. 2021, 22, bbaa307. 33230549 10.1093/bib/bbaa307 PMC8294548

[57] Y. Fang , L. Wang , C. Wan , Y. Sun , K. Van der Jeught , Z. Zhou , T. Dong , K. M. So , T. Yu , Y. Li , H. Eyvani , A. B. Colter , E. Dong , S. Cao , J. Wang , B. P. Schneider , G. E. Sandusky , Y. Liu , C. Zhang , X. Lu , X. Zhang , J. Clin. Invest. 2021, 131, e140837. 32990678 10.1172/JCI140837 PMC7773365

[58] M. Kanehisa , S. Goto , Nucleic Acids Res. 2000, 28, 27. 10592173 10.1093/nar/28.1.27 PMC102409

[59] M. H. Saier Jr. , C. V. Tran , R. D. Barabote , Nucleic Acids Res. 2006, 34, D181. 16381841 10.1093/nar/gkj001 PMC1334385

[60] C. T. Hensley , A. T. Wasti , R. J. DeBerardinis , J. Clin. Invest. 2013, 123, 3678. 23999442 10.1172/JCI69600 PMC3754270

[61] Z. Zhang , H. Zhu , P. Dang , J. Wang , W. Chang , X. Wang , N. Alghamdi , A. Lu , Y. Zang , W. Wu , Y. Wang , Y. Zhang , S. Cao , C. Zhang , Nucleic Acids Res. 2023, 51, W180. 37216602 10.1093/nar/gkad444 PMC10320190

[62] Z. Zhou , K. Van der Jeught , Y. Fang , T. Yu , Y. Li , Z. Ao , S. Liu , L. Zhang , Y. Yang , H. Eyvani , M. L. Cox , X. Wang , X. He , G. Ji , B. P. Schneider , F. Guo , J. Wan , X. Zhang , X. Lu , Nat. Biomed. Eng. 2021, 5, 1320. 34725507 10.1038/s41551-021-00805-x PMC8647932

