# Supplementary material for: Inhibition of Glutamate‐to‐Glutathione Flux Promotes Tumor Antigen Presentation in Colorectal Cancer Cells
Source: Adv Sci (Weinh). 2024 Oct 31;12(1):2310308. doi: 10.1002/advs.202310308 (PMC11714253; doi:10.1002/advs.202310308)
Supplement: Supplementary file 1 — Supporting Information [file ADVS-12-2310308-s001.docx]

**Supporting Information**

**Inhibition of Glutamate-to-Glutathione Flux Promotes Tumor Antigen Presentation in Colorectal Cancer Cells**

*Tao Yu, Kevin Van der Jeught, Haiqi Zhu, Zhuolong Zhou, Samantha Sharma, Sheng Liu, Haniyeh Eyvani, Ka Man So, Naresh Singh, Jia Wang, George E. Sandusky, Yunlong Liu, Mateusz Opyrchal, Sha Cao, Jun Wan, Chi Zhang, Xinna Zhang*

**Inventory of Supporting Information**

- **Figures S1-S9 and Legends**
- **Supporting Tables**

**Table S1. Clinical information on CRC patient samples**

**Table S2. Network (module) information**

**Table S3. Genes involved in each module**

**Table S4. Antibody list**

**Table S5. Primer list**

**Figure S1**

**
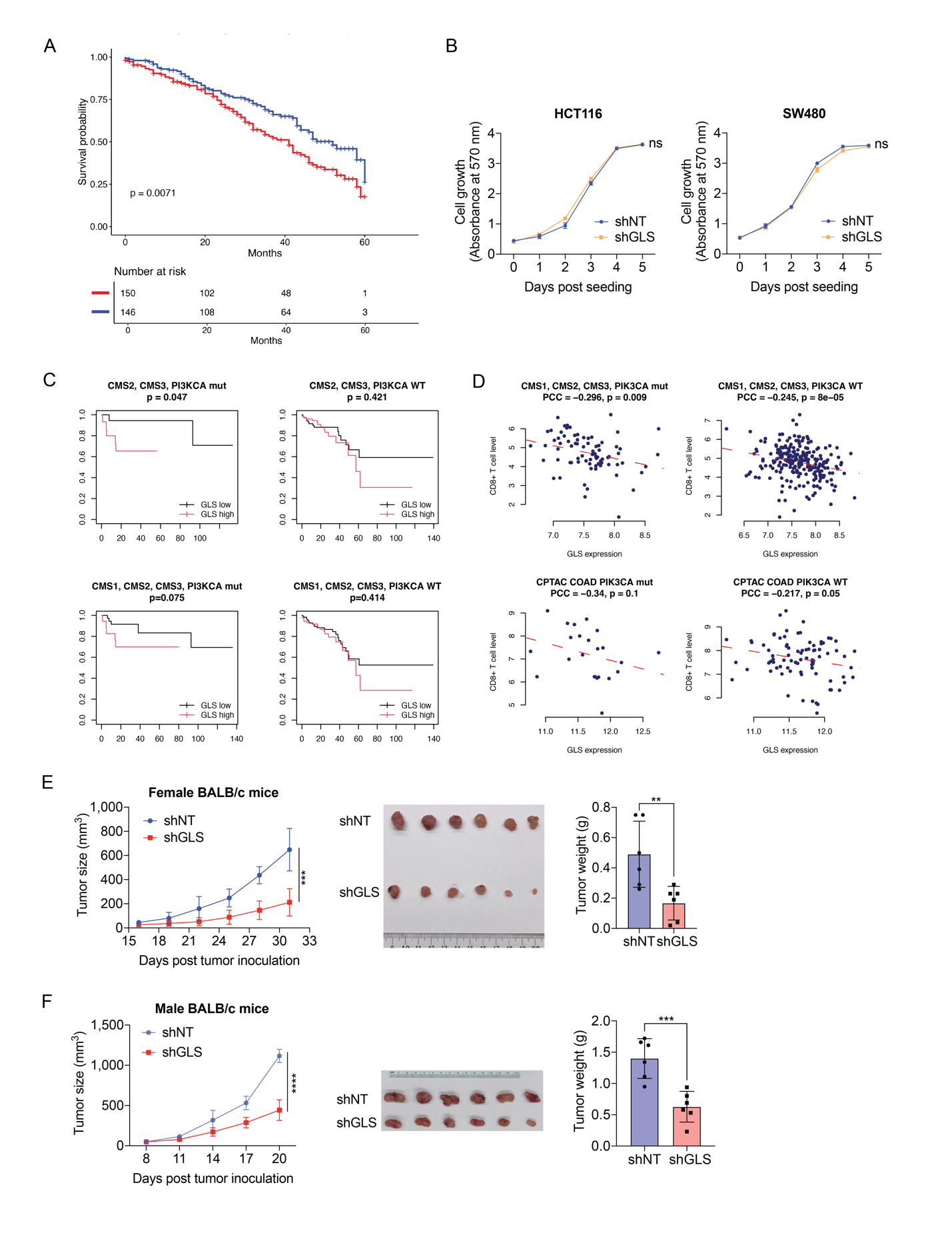
**

**Figure S1. Inhibition of GLS suppresses tumor growth in immunocompetent mice.**

(**A**) Kaplan-Meier survival curve with a 5-year cutoff from the GSE39582 (n = 505) dataset of CRC cases with high and low GLS expression (top and bottom 40%). (**B**) Cell proliferation of HCT116 and SW480 cells expressing control shRNA (shNT) or *GLS* shRNA (shGLS). The cell proliferation was determined using violet crystal staining at indicated time points. Data were analyzed using the unpaired two-tailed t-test for the absorbance at the endpoint and presented as mean ± SD (n=5). (**C**) Kaplan-Meier survival curve from the TCGA database of CRC cases with wildtype or mutant PI3KCA. (**D**) The correlations between GLS expression and CD8^+^ T cell level from both TCGA (Consensus Molecular Subtype classes 1-3) and CPTAC datasets. (**E,F**) CT26 cells expressing shNT or shGLS were inoculated subcutaneously into female (**E**) and male (**F**) BALB/c mice. The tumor image and tumor weights were taken at the endpoint. The tumor sizes were measured at indicated time points. Data were analyzed using the unpaired two-tailed t-test for the tumor sizes and weights at the endpoint and presented as mean ± SD (n=6).

**Figure S2**

**
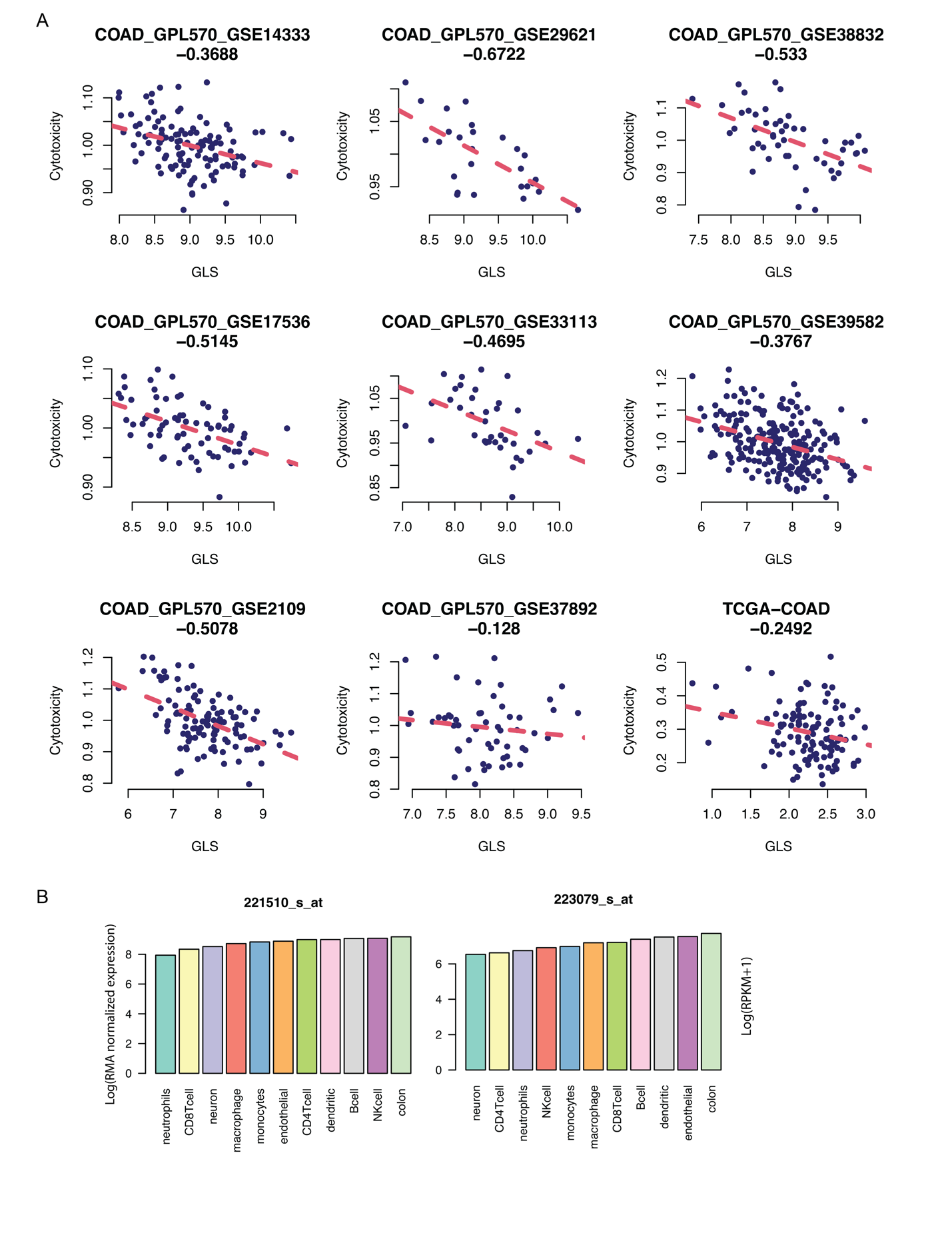
**

**Figure S2. The correlation analysis of the GLS expression with T cell cytotoxicity.**

(**A**) Correlation analysis between GLS expression level and T cell cytotoxicity in nine CRC datasets. (**B**) GLS expression levels in different cell types or tissues were analyzed using two GLS-specific probes in cell line data.

**Figure S3**

**
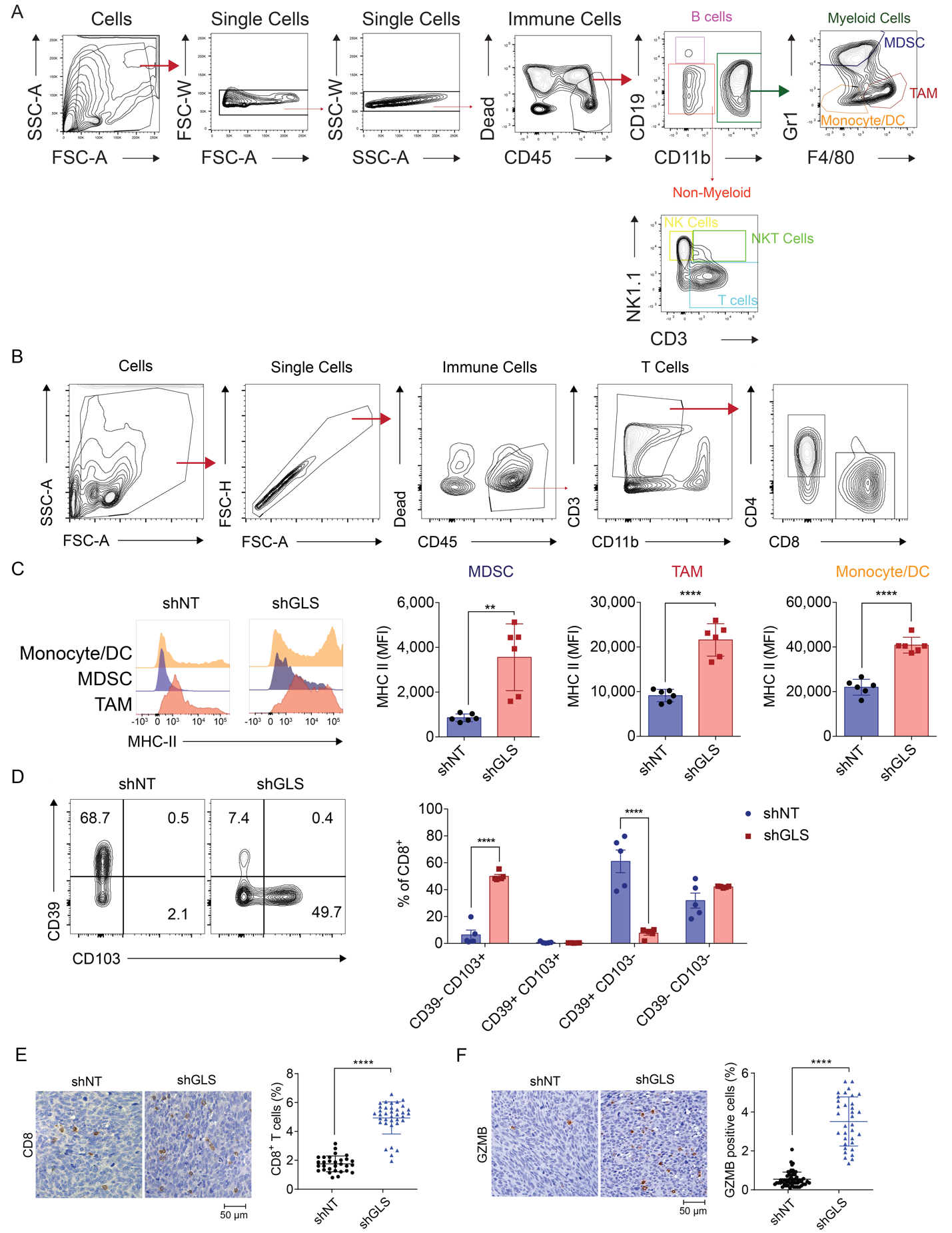
**

**Figure S3. Flow cytometry gating strategy, and the assessment of TILs in the TME.**

(**A**) Flow cytometry gating strategy to assess the total tumor microenvironment immune cell profiles. (**B**) Flow cytometry gating strategy to assess CD8^+^ T cell exhaustion, followed by the markers indicated in the main Figure 3. (**C**) Histogram overlay of the MHC class II (MHC-II) marker on the respective myeloid cell subpopulations with quantification. Data were analyzed using the unpaired two-tailed t-test (n=6; data displayed as mean ± SD). (**D**) Representative contour plots of CD39 and CD103 markers on CD8^+^ T cells with quantification. Data were analyzed using the unpaired two-tailed t-test (n=5; data displayed as mean ± SD). (**E,F**) Immunohistochemistry staining of CD8 (**E**) and granzyme B (GZMB) (**F**) in control or GLS-KD MC38 cells-derived tumors. The quantification was performed using ImageJ on around 30 images from each group. Data were analyzed using the unpaired two-tailed t-test and presented as mean ± SD.

**Figure S4**


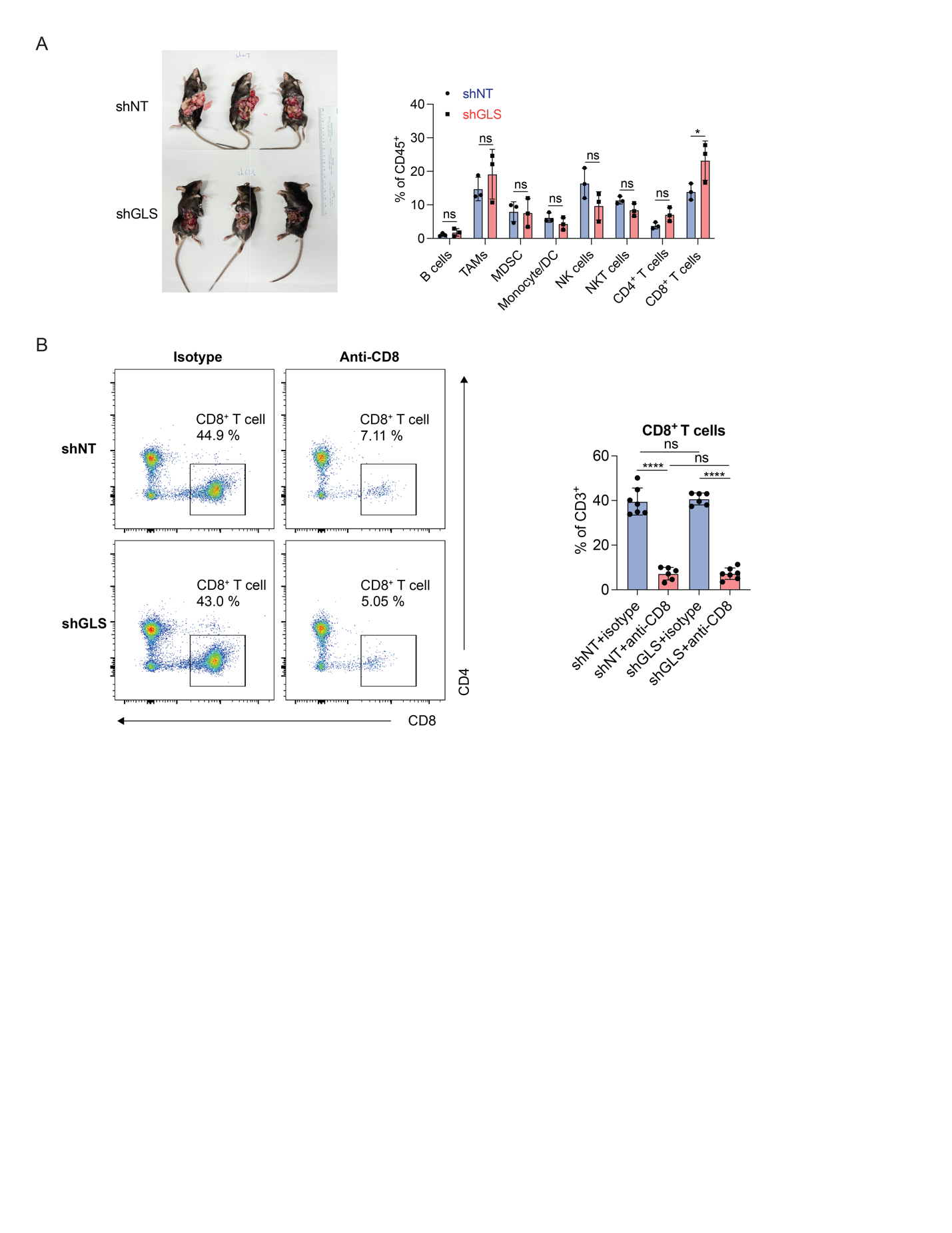


**Figure S4. Depletion of GLS in CRC cells alters the tumor immune microenvironment.**

(**A**) Flow cytometry analysis of tumor microenvironment changes in MC38 control and GLS-KD orthotopic tumors. Data were analyzed using two-way ANOVA and Sidak’s multiple comparisons test and presented as mean ± SD (n=3). (**B**) Representative flow cytometry plots and the quantification of the CD8^+^ T cell depletion from C57BL/6 mice inoculated subcutaneously with MC38 cells expressing shNT or shGLS and treated with the isotype or anti-CD8 antibody. Data were analyzed using one-way ANOVA and Sidak’s multiple comparisons test and displayed as mean ± SD (shNT+isotype, n=7; shNT+anti-CD8, n=6; shGLS+isotype, n=6; shGLS+anti-CD8, n=7).

**Figure S5**


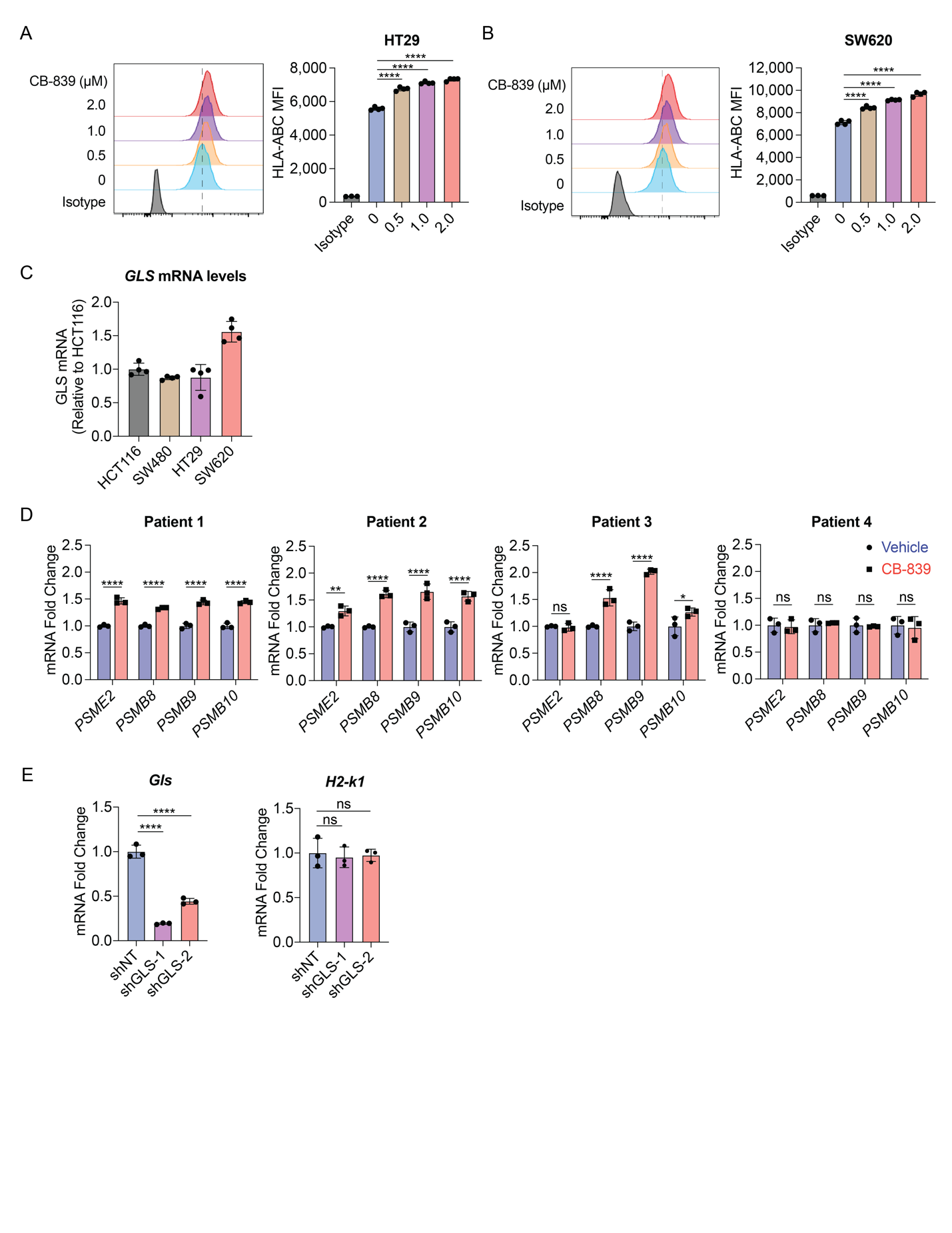


**Figure S5. Inhibition of GLS enhances tumor antigen presentation.**

(**A,B**) Cell surface HLA-A,B,C levels were determined using flow cytometry in human CRC cell lines HT29 (**A**) and SW620 (**B**) treated with CB-839 at indicated doses for 2 days. Data were analyzed using one-way ANOVA and Dunnett's multiple comparisons test and presented as mean ± SD (n=4). (**C**) GLS mRNA levels among different human CRC cells were determined by qPCR. (**D**) mRNA expression levels of immunoproteasome genes were determined using qPCR in the organoid cells from Figure 2C. Data were analyzed using two-way ANOVA and Sidak’s multiple comparisons test and presented as mean ± SD (n=3). (**E**) mRNA expression levels of *GLS* and *H2-k1* were determined using qPCR in control and GLS-KD MC38 cells. Data were analyzed using one-way ANOVA and Dunnett’s multiple comparisons test and presented as mean ± SD (n=3).

**Figure S6**

**
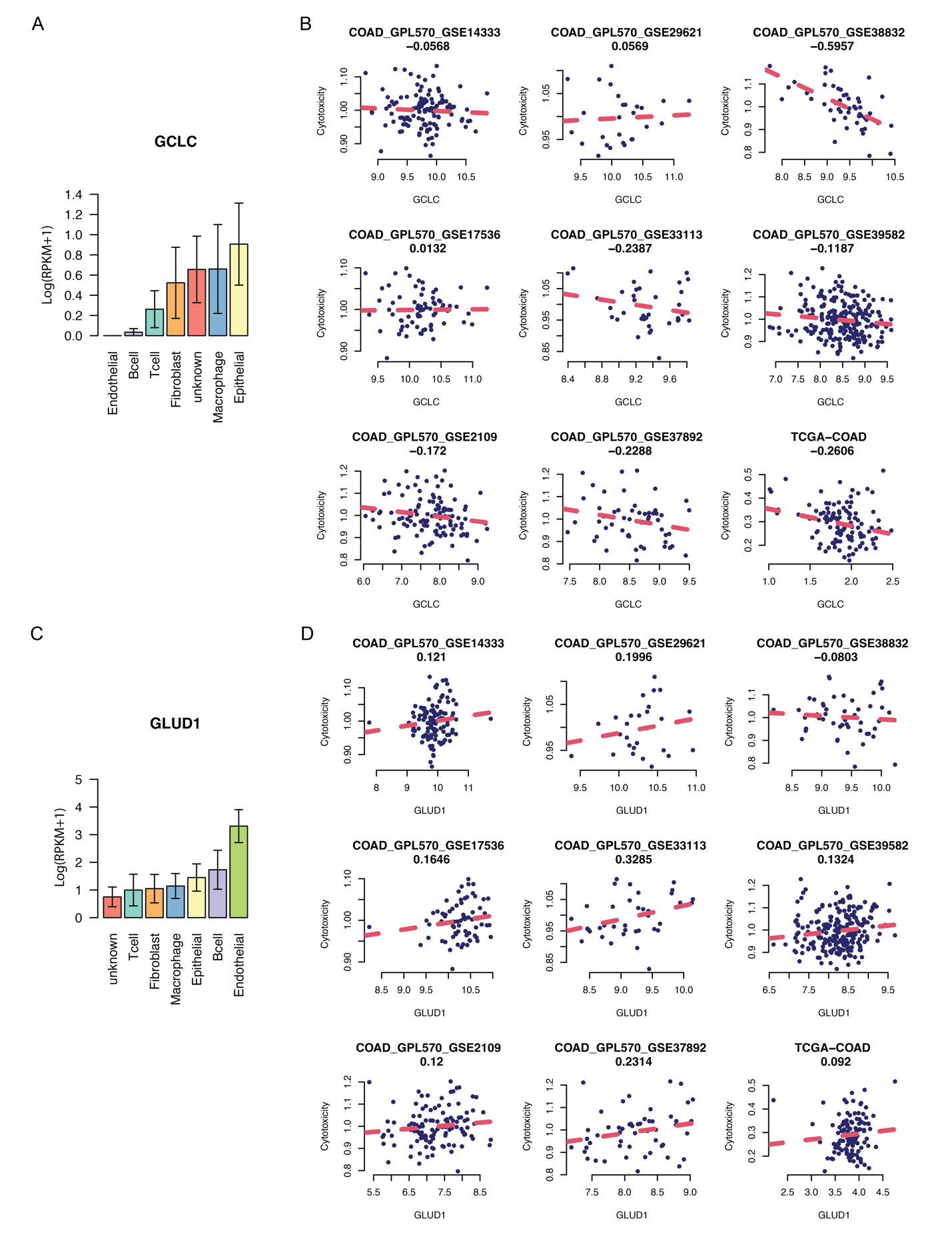
**

**Figure S6. The correlation analysis of the expression of GCLC and GLUD1, two key enzymes in the glutamine metabolic pathway, with T cell cytotoxicity.**

(**A**) GCLC expression levels in different cell types in scRNA-seq data of CRC samples. (**B**) Correlation analysis between GCLC expression level and T cell cytotoxicity in the nine CRC datasets. (**C**) GLUD1 expression levels in different cell types in scRNA-seq data of CRC samples. (**D**) Correlation analysis between GLUD1 expression level and T cell cytotoxicity in nine CRC datasets.

**Figure S7**

**
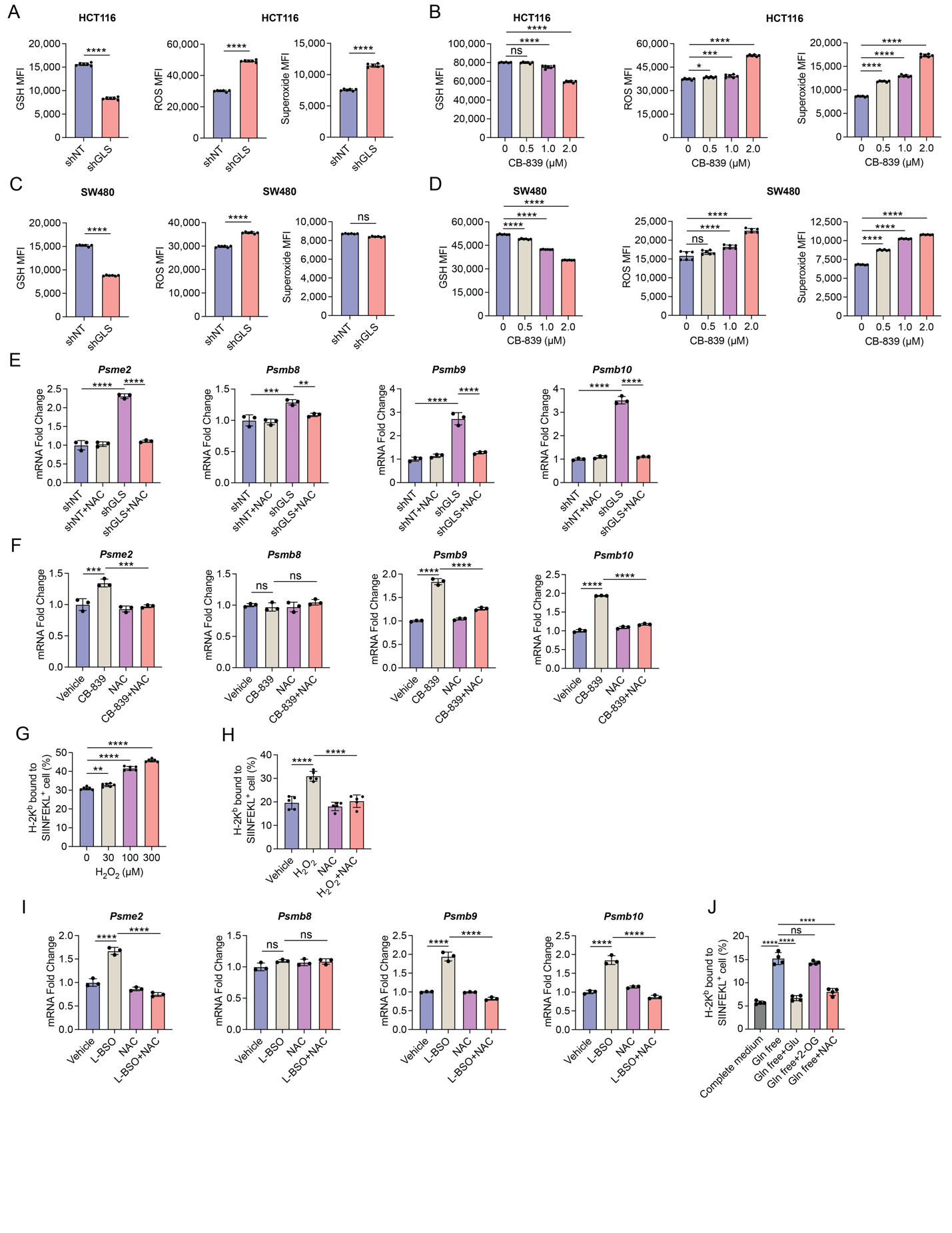
**

**Figure S7. ROS regulates tumor MHC-I-mediated antigen presentation.**

(**A**) GSH, ROS, and superoxide levels were determined by flow cytometry in control and GLS-KD HCT116 cells. Data were analyzed using the unpaired two-tailed t-test and presented as mean ± SD (n=6). (**B**) GSH, ROS, and superoxide levels were determined by flow cytometry in HCT116 cells treated with CB-839 at indicated doses. Data were analyzed using One-way ANOVA, and Dunnett's multiple comparisons test and presented as mean ± SD (n=6). (**C**) GSH, ROS, and superoxide levels were determined by flow cytometry in control and GLS-KD SW480 cells. Data were analyzed using the unpaired two-tailed t-test and presented as mean ± SD (n=6). (**D**) GSH, ROS, and superoxide levels were determined by flow cytometry in SW480 cells treated with CB-839 at indicated doses. Data were analyzed using One-way ANOVA, and Dunnett's multiple comparisons test and presented as mean ± SD (n=6). (**E**) mRNA expression levels of immunoproteasome genes were determined using qPCR in control and GLS-KD MC38 cells treated with or without NAC (5 mM). Data were analyzed using One-way ANOVA, and Tukey's multiple comparisons test and presented as mean ± SD (n=3). (**F**) mRNA expression levels of immunoproteasome genes were determined using qPCR in MC38 cells treated with CB-839 (2 μM) and/or NAC (5 mM) for 2 days. Data were analyzed using One-way ANOVA, and Tukey's multiple comparisons test and presented as mean ± SD (n=3). (**G**) H-2K^b^ mediated SIINFEKL (OVA) presentation levels in MC38-OVA cells treated with H_2_O_2_ at indicated doses were determined using flow cytometry. Data were analyzed using One-way ANOVA, and Dunnett's multiple comparisons test and presented as mean ± SD (n=6). (**H**) H-2K^b^ mediated SIINFEKL (OVA) presentation levels in MC38-OVA cells treated with H_2_O_2_ and/or NAC were determined using flow cytometry. Data were analyzed using One-way ANOVA, and Tukey's multiple comparisons test and presented as mean ± SD (n=5). (**I**) mRNA expression levels of immunoproteasome genes were determined using qPCR in MC38 cells treated with L-BSO (100 μM) and/or NAC (5 mM) for 2 days. Data were analyzed using One-way ANOVA, and Tukey's multiple comparisons test and presented as mean ± SD (n=3). (**J**) H-2K^b^ mediated SIINFEKL (OVA) presentation levels in MC38-OVA cells cultured with different metabolite modifications. Gln: glutamine; Glu: glutamate; 2-OG: 2-oxoglutarate; NAC: N-Acetyl Cysteine. Data were analyzed using one-way ANOVA, and Tukey's multiple comparisons test and presented as mean ± SD (n=4).

**Figure S8**

**
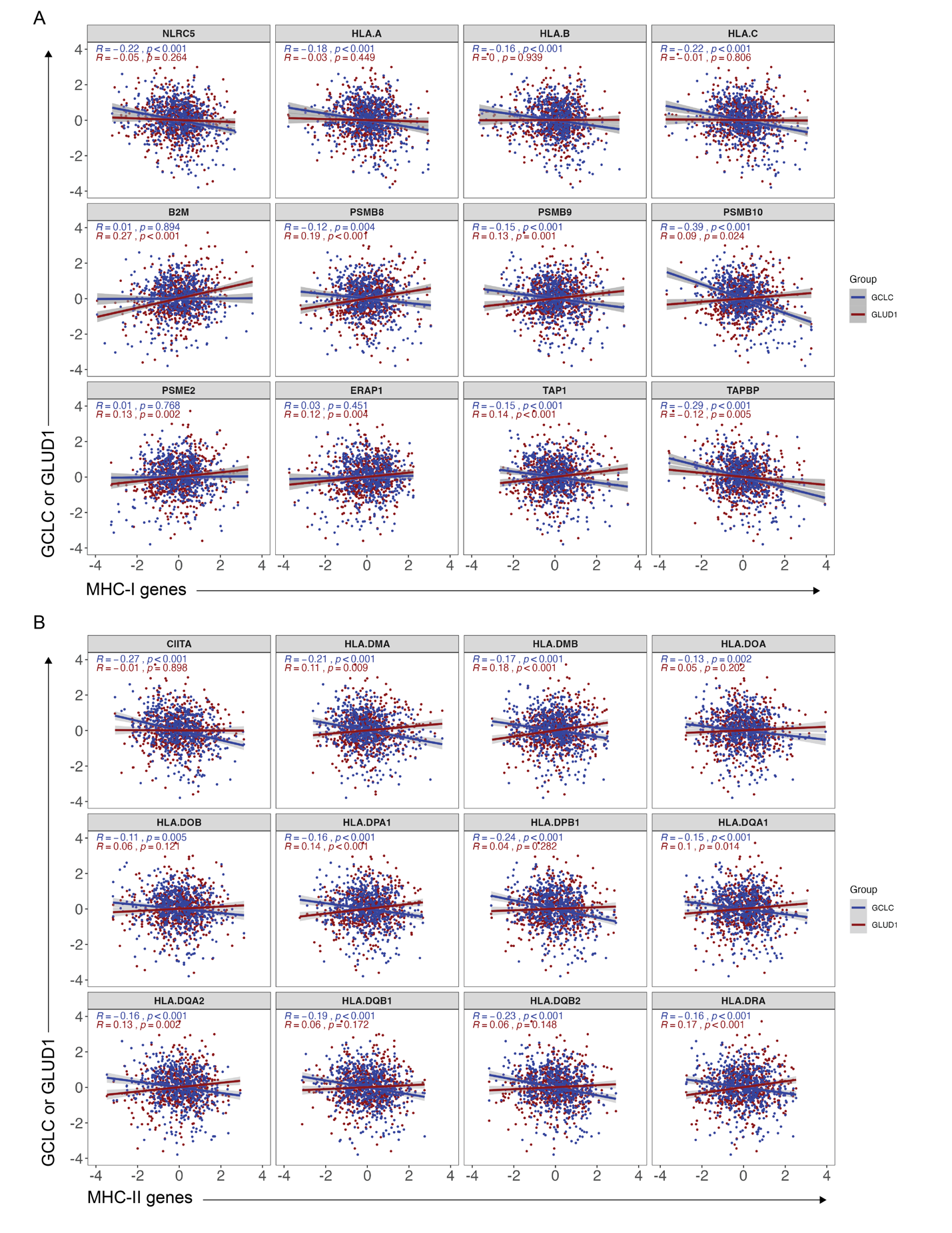
**

**Figure S8. The correlation analysis of the expression of GCLC and GLUD1, two key enzymes in the glutamine metabolic pathway, with MHC-I/II-associated genes in the TCGA CRC dataset.**

(**A**) Correlation analysis of *GCLC* or *GLUD1* with MHC-I-mediated antigen presentation genes from the Colorectal Adenocarcinoma (TCGA, PanCancer Atlas) dataset. (**B**) Correlation analysis of *GCLC* or *GLUD1* with MHC-II-mediated antigen presentation genes from the Colorectal Adenocarcinoma (TCGA, PanCancer Atlas) dataset.

**Figure S9**

A


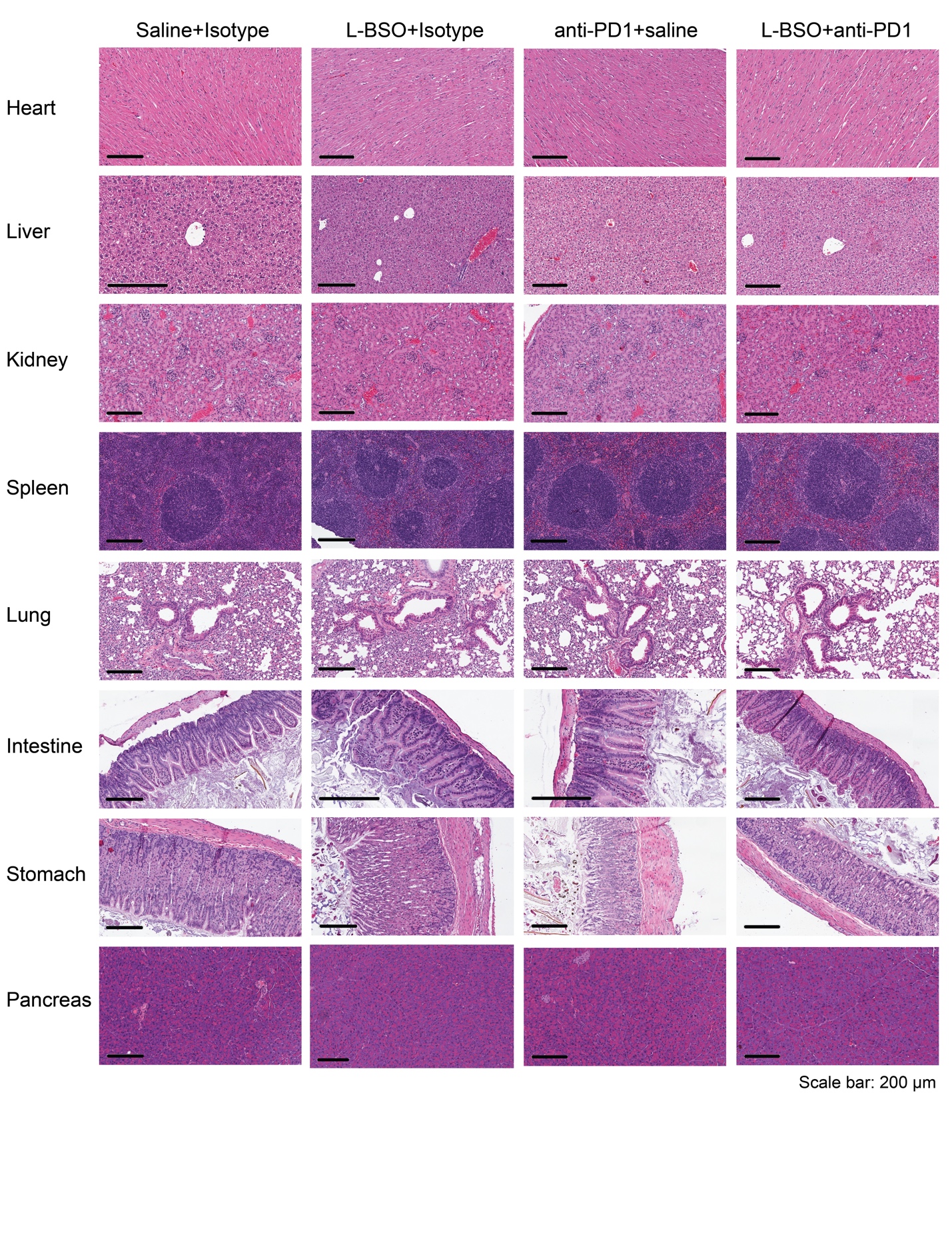


B

| **Groups** | **Tissue** | **Kidney** | **Liver** | **Lung** | **Pancreas** | **Spleen** | **Stomach** | **Heart** | **Intestine** |
| --- | --- | --- | --- | --- | --- | --- | --- | --- | --- |
| Saline + isotype | Positivity | Neg | Neg | Neg | Neg | Neg | Neg | Neg | Neg |
| L-BSO + isotype | Positivity | Neg | Neg | Neg  (Tumor near bronchiole in one sample) | Neg | Neg | Neg | Neg | Neg |
| Anti-PD-1 + saline | Positivity | Neg | Neg | Neg | Neg | Enlarged | Neg | Neg | Neg |
| L-BSO +  anti-PD-1 | Positivity | Neg | Neg | Neg | Neg | Neg | Neg | Neg | Neg |

**Figure S9. Assessment of organ toxicity from L-BSO and anti-PD-1 antibody-treated mice.**

(**A**) H&E staining of different organs harvested from the mice in the experiment of the main Figure 7I. One representative visual spot image was shown for each organ from the four groups. (**B**) Organ toxicity in the mice was assessed by a pathologist. Neg: negative for organ toxicity.

**Table S1. Clinical information on CRC patient samples**

| **Patient No.** | **Identification** | **Sex** | **Age** | **Tumor site** | **Histologic Type** | **Diagnosis** |
| --- | --- | --- | --- | --- | --- | --- |
| Patient 1 | UHB2008-03 | M | 61 | Cecum | Not applicable | Colon adenocarcinoma |
| Patient 2 | UHB2007-08 | M | 61 | Liver | Metastatic adenocarcinoma (colorectal primary) | Rectal adenocarcinoma |
| Patient 3 | UHB2010-19 | F | 67 | Jejunum | Adenocarcinoma | Colon adenocarcinoma |
| Patient 4 | EHB2008-05 | M | 63 | Sigmoid colon | Adenocarcinoma | Colon adenocarcinoma |

**Table S2. Network (module) information**

| **M_id** | **M_name** | **C_in** | **C_in_name** | **C_out** | **C_out_name** | **SL** | **SM_id** |
| --- | --- | --- | --- | --- | --- | --- | --- |
| M_1 | Glucose import -> Glucose | C00267_i | Glucose import | C00267 | Glucose | E->C | 12 |
| M_2 | Glucose -> G3P | C00267 | Glucose | C00118 | G3P | C | 1 |
| M_3 | G3P -> 3PD | C00118 | G3P | C00197 | 3PG | C | 1 |
| M_4 | 3PD -> Pyruvate_Cyto | C00197 | 3PG | C00022_c | Pyruvate_Cyto | C | 1 |
| M_5 | G3P -> PRPP | C00118 | G3P | C00119 | PRPP | C | 1 |
| M_6 | 3PG -> Serine | C00197 | 3PG | C00065 | Serine | C | 1 |
| M_7 | Pyruvate_Cyto -> Lactate | C00022_c | Pyruvate_Cyto | C00186 | Lactate | C | 1 |
| M_8 | Lactate -> Lactate_out | C00186 | Lactate | C00186_o | Lactate_out | C | 12 |
| M_9 | Pyruvate_Cyto -> Acetyl-CoA | C00022_c | Pyruvate_Cyto | C00024 | Acetyl-CoA | M | 1 |
| M_10 | glutamate_import -> Glutamate_Cyto | C00000 | glutamate_in2 | C00025_c | Glutamate_Cyto | C | 12 |
| M_11 | glutamine_import -> Glutamine_Cyto | C00000 | glutamine_in2 | C00064_c | Glutamine_Cyto | C | 12 |
| M_12 | glutamate_from other reactions -> Glutamate_Cyto | C00000 | glutamate_in | C00025_c | Glutamate_Cyto | C | 12 |
| M_13 | Glutamine_from other reactions -> Glutamine_Cyto | C00000 | Glutamine_in | C00064_c | Glutamine_Cyto | C | 12 |
| M_14 | Glutamine_Cyto -> other amino acids/metabolites | C00064_c | Glutamine_Cyto | C00000 | glutamine_out | C | 12 |
| M_15 | Glutamate_Cyto -> other amino acids/metabolites | C00025_c | Glutamate_Cyto | C00000 | glutamate_out | C | 12 |
| M_16 | Glutamine_Cyto -> Glutamine_Mito | C00064_c | Glutamine_Cyto | C00064 | Glutamine_Mito | C->M | 12 |
| M_17 | Glutamine_Mito -> Glutamate_Mito | C00064 | Glutamine_Mito | C00025 | Glutamate_Mito | M | 8 |
| M_18 | Glutamate_Mito -> Glutamate_Cyto | C00025 | Glutamate_Mito | C00025_c | Glutamate_Cyto | M->C | 12 |
| M_19 | Oxaloacetate+Glutamate_Mito-> 2OG_Mito+Asparate | C00036+C00025 | Oxaloacetate+Glutamate_Mito | C00026+C00049 | 2OG_Mito+Asparate | M | 1 |
| M_20 | Acetyl-CoA+Oxaloacetate -> Citrate_Mito | C00024+C00036 | Acetyl-CoA+Oxaloacetate | C00158 | Citrate_Mito | M | 1 |
| M_21 | Citrate_Mito -> Isocitrate | C00158 | Citrate_Mito | C00311 | Isocitrate | M | 1 |
| M_22 | Isocitrate -> 2OG_Mito | C00311 | Isocitrate | C00026 | 2OG_Mito | M | 1 |
| M_23 | 2OG_Succinyl-CoA | C00026 | 2OG_Mito | C00091 | Succinyl-CoA | M | 1 |
| M_24 | Succinyl-CoA_Succinate | C00091 | Succinyl-CoA | C00042 | Succinate | M | 1 |
| M_25 | Succinate_Malate | C00042 | Succinate | C00149 | Malate | M | 1 |
| M_26 | Malate_Oxaloacetate | C00149 | Malate | C00036 | Oxaloacetate | M | 1 |
| M_27 | Glutamate_Mito+L-Cysteine -> GSH | C00025+C00097 | Glutamate_Mito+L-Cysteine | C00051 | GSH | M | 2 |
| M_28 | Glutamate_Mito-> 2OG_Mito | C00025 | Glutamate_Mito | C00026 | 2OG_Mito | M | 1 |
| M_29 | GSH_in -> GSH | C00051_i | GSH_in | C00051 | GSH | M | 2 |
| M_30 | GSH -> GSH_out | C00051 | GSH | C00051_o | GSH_out | M | 2 |
| M_31 | GSH -> GSSG | C00051 | GSH | C00127 | GSSG | M | 2 |

**Table S3. Genes involved in each module**

| M_1 | SLC2A1, SLC2A2, SLC2A3, SLC2A4 |
| --- | --- |
| M_2 | ADPGK, G6PC, G6PC2, G6PC3, GALM, GCK, HK1, HK2, HK3, HKDC1, ALDOA, ALDOB, ALDOC, FBP1, FBP2, GPI, PFKL, PFKM, PFKP, TPI1 |
| M_3 | BPGM, CL640, COQ10D1, COQ2, GAPDH, GAPDHS, MSA1, PGAM1, PGAM2, PGAM4, PGK1, PGK2, PHB, PPT |
| M_4 | BPGM, CL640, COQ10D1, COQ2, ENO1, ENO2, ENO3, HIPER1, MINPP1, MINPP2, MIPP, MSA1, PCK1, PCK2, PGAM1, PGAM2, PGAM4, PHB, PKLR, PKM, PPT |
| M_5 | 6PGD, ALDOA, ALDOB, ALDOC, FBP1, FBP2, G6PD, G6PD1, GPI, H6PD, PFKL, PFKM, PFKP, PGD, PGLS, PGM1, PGM2, PRPS1, PRPS1L1, PRPS2, RBKS, RPE, RPEL1, RPIA, TALDO1, TKT, TKTL1, TKTL2 |
| M_6 | PHGDH, PSAT1, PSPH |
| M_7 | LDHA, LDHAL6A, LDHAL6B, LDHB, LDHC, LDHD |
| M_8 | SLC16A1, SLC16A3, SLC16A4 |
| M_9 | MPC1, MPC2, DLAT, DLD, DLDD, DLDH, E3, GCSL, LAD, PDHA1, PDHA2, PDHB, PHE3 |
| M_10 | ALDH4A1, OPLAH, GPT2, AGXT, AGXT2, GGT6, GGT1, GGT7, GGT5, OAT, TAT, BCAT1, BCAT2, AADAT, AASS, GGH, NAALAD2, NAALADL1, FOLH1 |
| M_11 | PPAT, GATC, PET112, QRSL1 |
| M_12 | SLC1A1, SLC1A2, SLC1A3, SLC1A5, SLC1A6, SLC1A7, SLC17A6, SLC17A8, SLC17A7, SLC7A11 |
| M_13 | SLC1A5, SLC38A1, SLC38A2 |
| M_14 | NADSYN1, CTPS1, CTPS2, CAD, ASNS, GFPT1, GFPT2, GMPS, CCBL2, CCBL1, QARS, PFAS, KYAT3, KYAT1 |
| M_15 | ALDH18A1, NAGS, GCLC, FPGS, PPAT, FTCD, GATC, PET112, QRSL1, GGT6, GGT1, GGT7, GGT5, EARS2, EPRS, TAT |
| M_16 | SLC1A5 |
| M_17 | GLS, GLS2, GLUL |
| M_18 | SLC25A22, SLC25A18 |
| M_19 | GOT2, GOT1, GOT1L1 |
| M_20 | ACLY, CS |
| M_21 | ACO1, ACO2 |
| M_22 | IDH1, IDH2, IDH3A, IDH3B, IDH3G |
| M_23 | OGDH, DLST |
| M_24 | SUCLG2 |
| M_25 | SDHA, FH |
| M_26 | MDH1 |
| M_27 | GCLC, GSS |
| M_28 | GLUD1, GOT1, GOT2, GPT |
| M_29 | GGT1, GGT5, GGT6, GGT7, GLO1, HAGH, HAGHL |
| M_30 | GSTA1, GSTA2, GSTA3, GSTA4, GSTA5, GSTK1, GSTM1, GSTM2, GSTM3, GSTM4, GSTM5, GSTO1, GSTO2, GSTP1, GSTT1, GSTT2, GSTT2B, LTC4S, MGST1, MGST2, MGST3 |
| M_31 | GPX1, GPX2, GPX3, GPX4, GPX5, GPX6, GPX7, GPX8, TXNDC12 |

**Table S4. Antibody list**

| **Antibody** | **Source** | **Catalog** |
| --- | --- | --- |
| PE/Cyanine7 anti-mouse CD3 (clone 17A2) | BioLegend | 100220 |
| Alexa Fluor 700 anti-mouse CD4 (clone GK1.5) | BioLegend | 100430 |
| APC/Cyanine7 anti-mouse CD8a (clone 53-6.7) | BioLegend | 100714 |
| Brilliant Violet 605 anti-mouse CD45 (clone 30-F11) | BioLegend | 103139 |
| Brilliant Violet 650™ anti-mouse/human CD11b (clone M1/70) | BioLegend | 101239 |
| PE anti-mouse/human CD11b (M1/70) | BioLegend | 101208 |
| FITC anti-mouse Ly-6G/Ly-6C (Gr-1) (clone RB6-8C5) | BioLegend | 108406 |
| Brilliant Violet 650™ anti-mouse CD19 (clone 6D5) | BioLegend | 115541 |
| PerCP/Cyanine5.5 anti-mouse F4/80 (clone BM8) | BioLegend | 123128 |
| APC anti-mouse CD25 (3C7) | BioLegend | 101910 |
| Brilliant Violet 785™ anti-mouse NK-1.1 (PK136) | BioLegend | 108749 |
| Brilliant Violet 421™ anti-mouse I-A/I-E (clone M5/114.15.2) | BioLegend | 107631 |
| R-PE labelled Pro-5 MHC Pentamer H-2kb KSPWFTTL (GP70) | Proimmune | F828-2B-D |
| BV650 Mouse Anti-Mouse Ly-108 | BD Biosciences | 740628 |
| Brilliant Violet 421™ anti-mouse CD279 (PD-1) Antibody | BioLegend | 135217 |
| Brilliant Violet 711™ anti-mouse CD366 (Tim-3) Antibody | BioLegend | 134021 |
| APC TOX Antibody, anti-human/mouse | Miltenyi Biotec | 130-118-335 |
| PE/Dazzle™ 594 anti-mouse CD223 (LAG-3) Antibody | BioLegend | 125223 |
| FITC anti-mouse/human CD44 Antibody | BioLegend | 103005 |
| Alexa Fluor® 488 anti-mouse CD38 Antibody | BioLegend | 102714 |
| APC anti-mouse CD39 Antibody | BioLegend | 143809 |
| APC anti-mouse CD62L Antibody | BioLegend | 104412 |
| Brilliant Violet 421™ anti-mouse CD103 Antibody | BioLegend | 121421 |
| Brilliant Violet 785™ anti-mouse IFN-γ Antibody | BioLegend | 505837 |
| Brilliant Violet 711™ anti-mouse TNF-α Antibody | BioLegend | 506349 |
| PerCP/Cyanine5.5 anti-human/mouse Granzyme B Recombinant Antibody | BioLegend | 372212 |
| APC anti-mouse H-2Kb bound to SIINFEKL (clone 25-D1.16) | BioLegend | 141606 |
| PE anti-human HLA-A,B,C Antibody | BioLegend | 311406 |
| eBioscience™ Fixable Viability Dye eFluor™ 506 | Invitrogen | 65-0866-14 |
| Anti-CD8α antibody | Cell Signaling Technology | 98941S |
| Anti-Granzyme B antibody | Cell Signaling Technology | 46890S |
| Anti-Glutaminase antibody | Abcam | ab93434 |
| Anti-Proteasome 20S LMP7 antibody | Abcam | ab3329 |
| Anti-Proteasome 20S LMP2 antibody | Abcam | ab184172 |
| Anti-PSMB10/MECL1 antibody | Abcam | ab183506 |
| Anti-PSME2 antibody | Abcam | ab183727 |
| Anti-Phospho-Stat1 (Ser727) antibody | Cell Signaling Technology | 8826S |
| Anti-Stat1 antibody | Cell Signaling Technology | 14994S |
| Anti-IRF-1 antibody | Cell Signaling Technology | 8478S |
| Ovalbumin antibody | Santa Cruz Biotechnology | sc-65984 |
| Anti-Beta-Actin Antibody | Abgent | AM1829B |
| mouse anti-rabbit IgG-HRP | Santa Cruz Biotechnology | sc-2357 |
| m-IgGκ BP-HRP | Santa Cruz Biotechnology | sc-516102 |
| 4Plus biotinylated goat anti-rabbit IgG | BIOCARE | GR602H |
| rat IgG2a isotype control (clone 2A3) | Bio X Cell | BE0089 |
| Anti-mouse PD-1 antibody (clone RMP1-14) | Bio X Cell | BE0146 |

**Table S5. Primer list**

| **Gene** | **Primer sequence** |
| --- | --- |
| Human *PSME2* F | 5’- GCAAGAGGACTCCCTCAATGT-3’ |
| Human *PSME2* R | 5’-CTTCTGGCTTAACCAGGGCA-3’ |
| Human *PSMB8* F | 5’-GCAGGCTGTACTATCTGCGAA-3’ |
| Human *PSMB8* R | 5’-AGAGCCGAGTCCCATGTTCAT-3’ |
| Human *PSMB9* F | 5’-GGTTCTGATTCCCGAGTGTCT-3’ |
| Human *PSME9* R | 5’-CAGCCAAAACAAGTGGAGGTT-3’ |
| Human *PSMB10* F | 5’-TCCTTCGAGAACTGCCAAAGA-3’ |
| Human *PSME10* R | 5’-ATCGTTAGTGGCTCGCGTATC-3’ |
| Human *ACTB* F | 5’-AGTGTGACGTGGACATCCGCAAAG-3’ |
| Human *ACTB* R | 5’-ATCCACATCTGCTGGAAGGTGGAC-3’ |
| Mouse *Gls* F | 5’-CTACAGGATTGCGAACATCTGAT-3’ |
| Mouse *Gls* R | 5’-ACACCATCTGACGTTGTCTGA-3’ |
| Mouse *Psme2* F | 5’-GAGAAGCCCGAAAACAGGTG-3’ |
| Mouse *Psme2* R | 5’-AGAGCTGACTCAGGGATATGATT-3’ |
| Mouse *Psmb8* F | 5’-ATGGCGTTACTGGATCTGTGC-3’ |
| Mouse *Psmb8* R | 5’-CGCGGAGAAACTGTAGTGTCC-3’ |
| Mouse *Psmb9* F | 5’-CATGAACCGAGATGGCTCTAGT-3’ |
| Mouse *Psmb9* R | 5’-TCATCGTAGAATTTTGGCAGCTC-3’ |
| Mouse *Psmb10* F | 5’-GAGGAATGCGTCCTTGGAACA-3’ |
| Mouse *Psmb10* R | 5’- CACAACCGAATCGTTAGTGGC-3’ |
| Mouse *H2-k1* F | 5’-ACCAGCAGTACGCCTACGA-3’ |
| Mouse *H2-k1* R | 5’-AACCAGAACAGCAACGGTCG-3’ |
| Mouse *Gaphd* F | 5’-AACGACCCCTTCATTGAC-3’ |
| Mouse *Gapdh* R | 5’-TCCACGACATACTCAGCAC-3’ |
